# Weak coupling between intracellular feedback loops explains dissociation of clock gene dynamics

**DOI:** 10.1371/journal.pcbi.1007330

**Published:** 2019-09-12

**Authors:** Christoph Schmal, Daisuke Ono, Jihwan Myung, J. Patrick Pett, Sato Honma, Ken-Ichi Honma, Hanspeter Herzel, Isao T. Tokuda

**Affiliations:** 1 Department of Mechanical Engineering, Ritsumeikan University, Kusatsu, Japan; 2 Institute for Theoretical Biology, Charité - Universitätsmedizin Berlin, Berlin, Germany; 3 Institute for Theoretical Biology, Humboldt Universität zu Berlin, Berlin, Germany; 4 Department of Neuroscience II, Research Institute of Environmental Medicine, Nagoya University, Nagoya, Japan; 5 Laboratory of Braintime, Shuang Ho Hospital, Taipei Medical University, New Taipei City, Taiwan; 6 Graduate Institute of Mind, Brain, and Consciousness, Taipei Medical University, Taipei, Taiwan; 7 Graduate Institute of Medical Sciences, Taipei Medical University, Taipei, Taiwan; 8 TMU Research Center of Brain and Consciousness, Shuang Ho Hospital, Taipei Medical University, New Taipei City, Taiwan; 9 Computational Neuroscience Unit, Okinawa Institute of Science and Technology, Okinawa, Japan; 10 Department of Chronomedicine, Hokkaido University Graduate School of Medicine, Sapporo, Japan; Université Libre de Bruxelles, BELGIUM

## Abstract

Circadian rhythms are generated by interlocked transcriptional-translational negative feedback loops (TTFLs), the molecular process implemented within a cell. The contributions, weighting and balancing between the multiple feedback loops remain debated. Dissociated, free-running dynamics in the expression of distinct clock genes has been described in recent experimental studies that applied various perturbations such as slice preparations, light pulses, jet-lag, and culture medium exchange. In this paper, we provide evidence that this “presumably transient” dissociation of circadian gene expression oscillations may occur at the single-cell level. Conceptual and detailed mechanistic mathematical modeling suggests that such dissociation is due to a weak interaction between multiple feedback loops present within a single cell. The dissociable loops provide insights into underlying mechanisms and general design principles of the molecular circadian clock.

## Author summary

Circadian clocks are endogenous pacemakers that generate gene expression oscillations with a period of approximately 24h. They enable an organism to anticipate daily changes in light and temperature and allow to align physiological properties to the most beneficial time around the solar day. The suprachiasmatic nucleus (SCN) of the hypothalamus is the master circadian pacemaker in mammals that coordinates peripheral clocks throughout the body and even encodes seasons. Gene expression oscillations of circadian clock genes in this master pacemaker have been shown to dissociate after perturbations of the system such as light pulses and jet-lag. The underlying mechanism remains unknown. We show that this dissociation may occur even within a single cell of the pacemaker. Data-driven mathematical modeling suggests that the dissociation relies upon a weak interaction between interlocked gene-regulatory feedback loops. Differential responses of these feedback loops to light perturbations is consistent with the concept of morning and evening oscillators.

## Introduction

Circadian clocks are omnipresent in almost all living organisms as a consequence of adaptation to 24 h environmental fluctuations, leading to convergent evolution across different kingdoms of life [1]. Interlocked transcriptional-translational feedback loops (TTFLs) have been identified as a common design principle for the generation of intracellular rhythms. A single negative feedback is a process, in which a gene product suppresses its own expression with a time delay. Interlocking between multiple loops may have both negative and positive effects on gene expressions. In mammals, the negative feedback system that is often considered as “primary-loop” [2] consists of the *Period* (*Per1*, *-2*, *-3*) and *Chryptochrome* (*Cry-1*, *-2*) as well as the bHLH-PAS transcription factors *Bmal1* (also *Arntl* or *Mop3*) and *Clock*. Heterodimers of CLOCK and BMAL1 proteins enhance the transcription of *Per* and *Clock* genes by binding to their E-box promoter elements. The products of these genes, PER and CLOCK proteins, antagonize the activatory effects of the CLOCK-BMAL1 heterodimers and thus close the delayed negative feedback loop. This feedback loop will be hereinafter referred to as the *Per loop*. In addition to the “primary-loop”, a nuclear receptor loop has been identified, involving *Ror* (*Ror* *α*, -*β*, -*γ*) as positive regulators of *Bmal1* and *RevErb* (*RevErbα*, -*β*) as negative regulators [3, 4]. Like *Per* and *Cry* genes, *RevErb* and *Ror* are transcriptionally activated by heterodimers of CLOCK and BMAL1. It has been shown by computational modeling [5] and confirmed by double-knockouts [6] that this loop plays an essential role in the rhythm generation. We will refer to this additional loop as the *Bmal-Rev loop*. It has been proposed that interlocking of such multiple loops contributes to the flexibility and robustness of the circadian system [7–10].

Complementary to experimental progress, mathematical modeling made a decisive contribution towards a better understanding of the design principles and complex dynamical behavior of the molecular circadian pacemakers across diverse organisms such as cynobacteria, fungus *Neurospora crassa*, plants, and mammals [5, 11–16] as well as regulatory modules downstream of the main clock [17, 18]. It has been commonly assumed that interaction of feedback loops confers robustness to molecular clock oscillations through phase- and frequency-locking of all component expressions. In the terminology of dynamical systems theory, the whole clock network constitutes a limit cycle oscillator, thereby all components form a periodic orbit of period *τ*, for which small perturbations from steady state dynamics decay with a characteristic time scale, that can be surprisingly long, even longer than 24 h. In the course of such “presumably transient” dynamics, individual components of the limit cycle oscillator may dissociate and could show different instantaneous periods, amplitude modulations and phase slips as they approach their steady state oscillations.

Recent experimental evidence shows that circadian rhythms of core clock genes dissociate at least transiently under certain conditions. *In situ* hybridization of mouse SCN revealed that circadian cycles of *mPer1* expression react more rapidly than those of *mCry1* expression to an advanced lighting schedule [19]. *Per1* and *Per2* mRNA rhythms in mouse SCN have been shown to exhibit a faster re-entrainment after a 6h jet-lag phase shift compared to those of *Bmal1*, *RevErbα* and *Dbp* [20]. In freely moving single-transgenic mice expressing either a *Bmal1-ELuc* or a *Per1-luc* reporter construct, re-entrainment to a new stable phase occurs at different time scales for two clock components *Bmal1* and *Per1* after application of a 9h light pulse at circadian time (CT, using the endogenous period *τ* as a reference) of 11.5h (*i.e.*, half an hour before subjective night) [21]. In addition to the behavioral studies, a dissociation of clock gene expressions has been observed among organotypic SCN slices. Measurements of bioluminescence signals in SCN slices carrying a single luciferase reporter construct revealed a significantly longer circadian period in PER2::LUC oscillations compared to *Bmal1-ELuc*, while the donor animals had identical locomotor activity periods [22]. From double-transgenic mice carrying both *Bmal1-ELuc* and *Per1-luc* reporter constructs, diverging phases were observed between the two differently colored luciferase reporters in the same slice. This leads again to significantly shorter *Bmal1-ELuc* periods across a three week long-term recording [21]. Dissociation of the two genes was observed in different reporter constructs that express luciferases with more distinct emission wavelengths [23]. Furthermore, phase response dynamics to timing of medium exchange were found to be different in *Bmal1-ELuc* and *Per2-SLR2* oscillations in cultured slices of the SCN [23]. While *Bmal1* showed a significant response to the medium exchange in neonatal mice [24], *Per2* did not [23].

Despite these experimental observations, existing mathematical models have not taken into account such perturbation-induced, long-lasting transient dissociation of clock genes, presumably involved in different feedback loops. We use data-driven conceptual and contextual modeling approaches to identify intracellular network topologies and parameter realizations that enable experimentally observed dissociation dynamics. Our theoretical model raises new questions on the design principles of interlocked molecular loops and proposes a possibility that the biological systems may utilize such dissociation of multiple feedback loops to differential responses to external environment.

## Results

### Surrogate data analysis suggests a dissociation of *Per1* and *Bmal1* dynamics at the single cell level

*Bmal1* and *Period* (*Per1*, *Per2*) clock genes have been shown to exhibit differential dynamics after perturbations such as light-pulses, jet-lag, *ex vivo* slice preparations and culture medium exchange. Although their dissociation has been suggested more directly by recent experiment [21], it remains unclear whether this dynamical dissociation occurs within an intra-cellular level. As discussed in detail in [21], two hypotheses can be considered. The first hypothesis $H_{0}^{\left(1\right)}$ states that the dissociation takes place within a single cell, i.e. the dynamics of different components within the same intra-cellular network dissociate (at least transiently). The second hypothesis $H_{0}^{\left(2\right)}$, on the other hand, assumes existence of two groups of cells, in which either Bmal1 or Per1 signal is predominant. In order to examine the two hypotheses, artificial time lapse movies, *i.e.*, surrogate data [25], have been created based on either of the two hypotheses and their oscillatory properties were further analyzed. Detailed procedure for generating the surrogate data can be found in Section *Materials and Methods*. S1 Fig illustrates various steps to generate the artificial time lapse movies.

**Fig 1:**
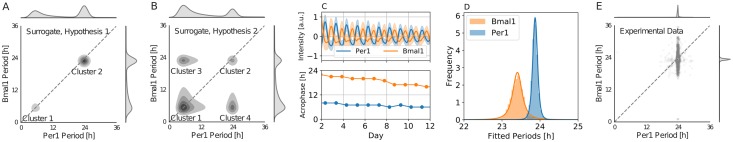
Statistical hypothesis testing indicates dissociation of *Bmal1-ELuc* and *Per1-luc* rhythms at the single cell level. A) *Gaussian* kernel density estimates in the bivariate graph of Bmal1 and Per1 oscillation periods, estimated by a Lomb Scargle analysis of surrogate time lapse movies, generated under hypothesis $H_{0}^{\left(1\right)}$, *i.e.*, dynamical dissociation at the single cell level. B) Same as panel (A) in case of hypothesis $H_{0}^{\left(2\right)}$, *i.e.*, randomly located cells with either Bmal1 or Per1 signal of different periods. In both panels, *N* = 150 cells have been randomly drawn. Signal intensities of 1, Bmal1 period of 23h, Per1 period of 24h, cell sizes *σ*_*G*_ = 0.0132 and noise strength of *σ*_*n*_ = 1 were used. See S1 Fig for an example. C) *Top:* Average values (bold line) and standard deviations (shaded areas) of *Per1-luc* (blue) and *Bmal1-ELuc* (orange) signals from a cultured SCN of double transgenic mice. *Bottom:* Times of oscillation peaks (acrophases) in the averaged *Per1-luc* (blue) and *Bmal1-ELuc* (orange) signals. Compared to *Per1-luc* signals, phase drift of *Bmal1-ELuc* in oscillation peak times can be observed, suggesting a shorter *Bmal1-ELuc* period. D) Histograms of pixel-wise oscillation periods in the *Per1-luc* (blue) and *Bmal1-ELuc* (orange) signals as determined by a Lomb Scargle periodogram analysis. Bold lines denote fits of a (non-central) Student’s t-distribution to the histogram data. The Student’s t-distribution has been preferred over normal distribution for its lower sensitivity to outliers [26]. Fitted parameters for the location (similar to the mean of a *Gaussian*) and scale parameter (similar to the standard deviation of a *Gaussian*) are ≈ 23.87 ± 0.06 h and ≈ 23.40 ± 0.13 h in case of *Per1-luc* and *Bmal1-Eluc* signals, respectively. E) Pixel-wise comparison of *Per1-luc* and *Bmal1-Eluc* periods as shown by a scatter plot (crosses) together with the corresponding kernel density estimates. The broader distribution of *Bmal1-ELuc* periods can be due to the lower SNR (signal-to-noise ratio) in comparison to the *Per1-luc* signal. Data analyzed in panels (C)-(E) correspond to the ones shown in Figure 5 of reference [21].

A pixel-wise analysis of oscillatory properties in the surrogate movie data reveals qualitative differences between the two hypotheses. In the case of surrogate data generated under hypothesis $H_{0}^{\left(1\right)}$, a pixel-wise comparison of Bmal1 and Per1 periods reveals two clusters in the corresponding bivariate graph, compare Fig 1A and S2 Fig. Pixel-wise time traces in cluster 1 have a non-circadian period close to zero in both, the Bmal1 and Per1 signals. This corresponds to pixels where no SCN cells are located and, thus, the dominant peak in the Lomb Scargle periodogram is located at periods much shorter than the circadian. Time traces in cluster 2 contain a dominant circadian component in both, the Bmal1 and Per1 signals, corresponding to pixels where SCN cells have been present. This situation changes qualitatively in the case of surrogate data generated under hypothesis $H_{0}^{\left(2\right)}$, where two additional clusters emerge, see Fig 1B. Cluster 1 still corresponds to time traces from pixels, where no significant circadian rhythm can be observed for both signals. Time traces in cluster 2 are again from pixels, where circadian periods have been detected in both Bmal1 and Per1 signals, *i.e.*, by chance a Per1 cell and a Bmal1 cell are closely located so that both signals spatially overlap with each other. In clusters 3 and 4, each pixel contains circadian component in either Bmal1 or Per1 signal, where no circadian rhythmicity is present in the other signal.

We next compared these qualitative features of the surrogate data with those of the corresponding experimental data. Bioluminescence recordings from *in vitro* SCN slices of neonatal double transgenic mice, expressing both *Per1-luc* and *Bmal1-ELuc* reporter constructs at the same time, have been therefore analyzed. Nearly anti-phasic relation between *Per1-luc* and *Bmal1-ELuc* oscillations can be observed in the detrended, averaged bioluminescence signals, see Fig 1C *top*. A closer inspection of the peak times of these averaged oscillatory signals reveals a steady phase drift between *Per1-luc* and *Bmal1-ELuc* peak times, compare Fig 1C *bottom*. This is reflected in the distributions of the pixel-wise Lomb Scargle period analysis, revealing a center of the distribution around ≈ 23.87 ± 0.06 h and ≈ 23.40 ± 0.13 h for *Per1-luc* and *Bmal1-Eluc* signals, respectively, see Fig 1D. These distributions are in good agreement with the previously reported shorter *Bmal1-ELuc* period in comparison to *Per1-luc* [21] or *Per2-SLR2* [23] signals in neonatal double transgenic mice. A pixel-wise comparison of *Bmal1-ELuc* and *Per1-luc* oscillation periods leads to a dominant single cluster in the bivariate graph, similar to the surrogate data as generated under hypothesis $H_{0}^{\left(1\right)}$, *i.e.*, dynamical dissociation at the single cell level, compare Fig 1A and 1E. The broader distribution of *Bmal1-ELuc* periods can be due to its lower SNR (signal-to-noise ratio) in comparison to the *Per1-luc* signals.

To conclude our statistical hypothesis testing, comparative analysis between experimental and surrogate data supports the idea that the dissociation of *Bmal1-ELuc* and *Per1-luc* signals in slices occurs at the single cellular level (hypothesis $H_{0}^{\left(1\right)}$).

### A conceptual model of two weakly coupled feedback loops explains differential responses of clock gene expression upon light perturbations

In the gene-regulatory network of circadian rhythms, Bmal1 has long been thought of as a major hub. Genetic knockout of Bmal1 leads to arrhythmicity in clock gene expression and behavioral rhythms under free-running conditions [27]. However, it has been shown that constitutive expression of BMAL1 (or BMAL2) in a Bmal1^-/-^ knockout mutant mice recovers rhythmic expression of *Per2* mRNA and behavioral activity at periods similar to WT oscillations, thus questioning the necessity of rhythmic BMAL1 protein oscillations with respect to proper clock functioning [28–30]. Furthermore, computational studies suggest, that both, the negative auto-regulatory Per loop as well as the Bmal-Rev negative feedback loop are able to oscillate autonomously [5, 31]. Persistence of circadian rhythmicity in transgenic rats overexpressing *mPer1*, although its responsiveness to light cycles was impaired, suggests alternative feedback loops that function without *mPer1* [32].

**Fig 2:**
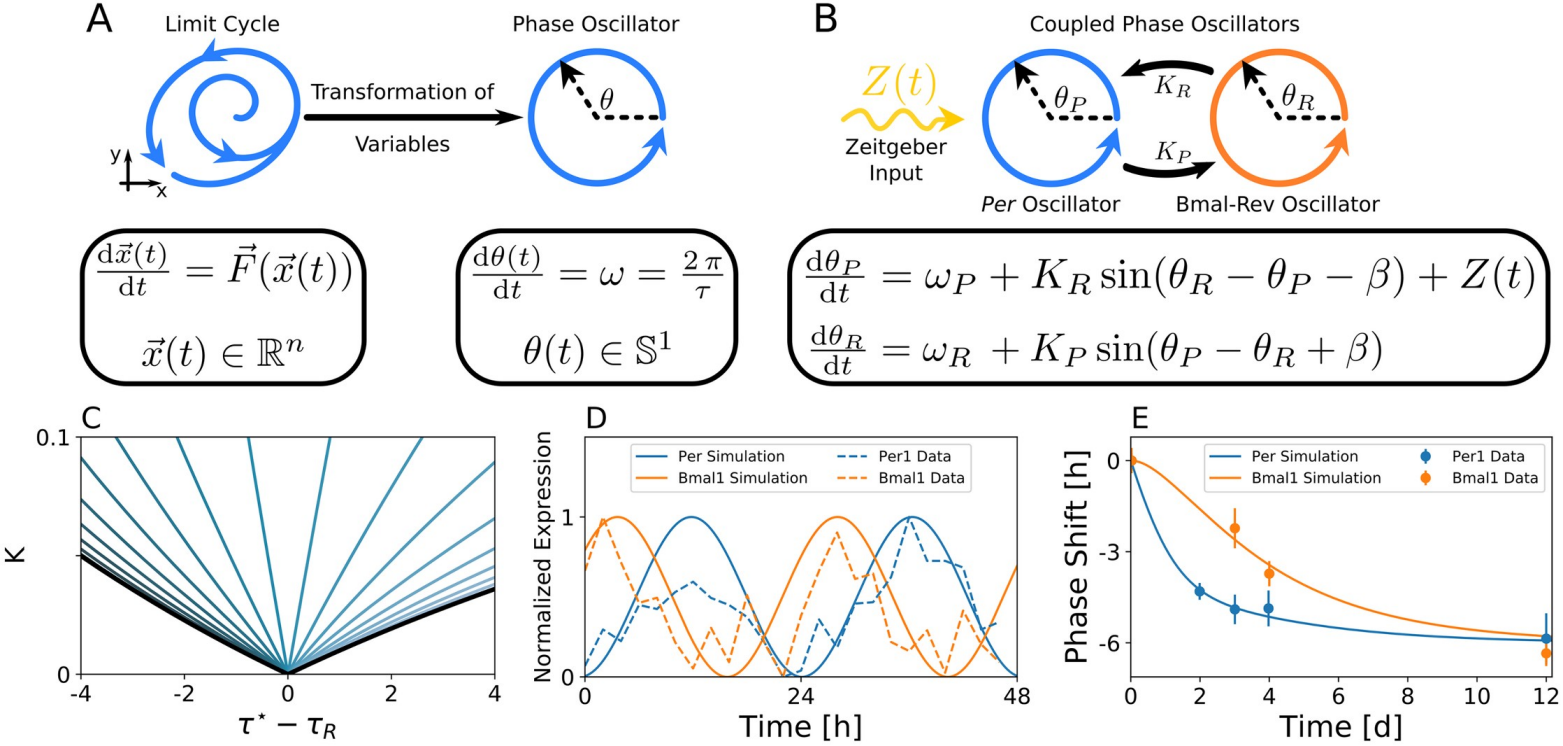
A light driven network of two coupled phase oscillators, representing the Per and Bmal-Rev loops, is able to reproduce experimental free-running and light perturbation data. A) Illustration of the phase oscillator concept. B) Schematic drawing of our conceptual model of light-driven, interlocked intra-cellular feedback loops. C) Isoclines of constant phase difference between the Per and the Bmal-Rev loops, color-coded for different values of *β*. Black lines denote the borders of synchronization between the Per and Bmal-Rev loops as determined by Eq (4) in Section *Materials and Methods*. Isoclines of constant Δ*θ*^⋆^ = −0.7*π*, corresponding to the experimentally observed phase difference of approximately 9 h between *Per1* and *Bmal1* expression in the time domain, are plotted and color-coded for different values of *β*, ranging from $\beta =-\Delta \theta^{⋆}-\frac{\pi }{2}$ to $\beta =-\Delta \theta^{⋆}+\frac{\pi }{2}$ in 20 equidistant steps. The experimentally observed oscillation period *τ*^⋆^ ≈ 24.53h and phase difference has been estimated by cosine fits to *Per1* and *Bmal1* circadian gene expressions from high-throughput transcriptome data of 48h length at 2h sampling intervals [33], see S3 Fig. General distributions of phase differences Δ*θ*^⋆^ within the range of synchronization between Per and Bmal-Rev loops for different values of *β* are depicted in S4 Fig D) Dynamics of experimentally observed *Per1* and *Bmal1* gene expression rhythms can be reproduced by the concpetual oscillator model. Bold lines denote the cosine of oscillation phases *θ*_*P*_(*t*) and *θ*_*R*_(*t*) of the corresponding Per and Bmal-Rev loop. E) Weakly coupled Per and Bmal-Rev loops can account for a faster re-entrainment of *Per1* compared to *Bmal1* gene expression oscillations after a 6h phase advancing jet-lag.

Motivated by these findings, we construct a conceptual model that considers interlocking of autonomously oscillating Per and Bmal-Rev intracellular feedback loops, based on the negative auto-regulation of Per and the composite negative feedback between Bmal1 and RevErb regulation, to describe transient dissociation of Bmal1 and Per1 dynamics. The dynamics of each loop is simplified by a phase oscillator, which reduces the high-dimensional limit cycle dynamics into a phase space of only a single variable, *i.e.*, the phase of oscillation *θ*. Fig 2A illustrates the concept of phase oscillator modeling. The phase dynamics of individual loops are assumed to be governed by intrinsic angular velocities *ω*_*P*_ and *ω*_*R*_, which are related to the internal period of the Per and Bmal-Rev loop by $\tau_{P}≔\frac{2\;\pi }{\omega_{P}}$ and $\tau_{R}≔\frac{2\;\pi }{\omega_{R}}$, respectively, and a sinusoidal interaction function. The underlying network topology and governing equations are depicted in Fig 2B, see also Eqs (1) and (2) of Section *Materials and Methods*. Parameters *K*_*R*_ and *K*_*P*_ determine the coupling strength between Per (*θ*_*P*_) and Bmal-Rev (*θ*_*R*_) loops as a function of their phase difference Δ*θ* ≔ *θ*_*P*_ − *θ*_*R*_. The stable phase difference Δ*θ*^⋆^ upon complete synchronization (vanishing period difference or phase-locking) of both loops can be flexibly adjusted by parameter *β*, see Eq (6) in *Materials and Methods*. *Per1* and *Per2* transcription has been shown to exhibit acute responses to light pulses during subjective night [34–36]. We thus assume that light resetting of the core clock network solely affects the Per loop but not the Bmal-Rev loop. For the sake of simplicity, we assume a sinusoidal *Zeitgeber* signal $Z(t)=z\;\text{sin}(\frac{2\;\pi }{T}t-\theta_{P}+\phi_{0})$, similar to previously published computational studies on entrainment of the mammalian circadian clock [37]. Here, *T* denotes the *Zeitgeber* period, while *z* determines the effective strength of the signal.

We aim to reproduce SCN expression profiles of *Per1* and *Bmal1* core clock gene oscillations under constant conditions as taken from a high-throughput transcriptome data set, recorded over a 48h period at a 2h sampling interval [33]. Under the assumption that clock genes are synchronized with a common oscillation period under steady state conditions, we estimate a steady state phase difference of approximately 9h or equivalently Δ*θ*^⋆^ ≈ −0.7*π* between the *Per1* and *Bmal1* mRNA rhythms at an oscillation period of *τ* ≈ 24.53h as revealed by a cosine fit to the corresponding experimental time series, see S3 Fig. For simplicity, we assume symmetric, equally strong, coupling strength between the Per and Bmal-Rev loops (*K*_*P*_ = *K*_*R*_ ≕ *K*) in both coupling directions. Under constant conditions (*z* = 0), the region of phase-locking between the Per and Bmal-Rev loops (also: synchronization regime) forms a triangular shape in the parameter plane of coupling-strength (*K*) and period-detuning (*τ*⋆ − *τ*_*R*_). The tip of the triangle touches the point of vanishing period differences on the abscissa, see Fig 2C, S4 Fig, and Eq (4) of Section *Materials and Methods*. Thus, for small coupling strength *K*, only small period detunings result in synchronized dynamics, while large coupling strength allows a synchronized state even for a larger detuning of periods. This is tantamount to the concept of *Arnold* tongues, describing entrainment regimes for externally forced endogenous oscillators [37, 38]. For any given parameter *β* that realizes dynamics with the experimentally observed steady state phase difference Δ*θ*^⋆^ ≈ −0.7*π* (which is given for all $-\Delta \theta^{⋆}-\frac{\pi }{2}<\beta <-\Delta \theta^{⋆}+\frac{\pi }{2}$), we can find an isocline of constant phase difference Δ*θ*^⋆^ within the synchronization regime as shown in Fig 2C. Each pair of parameters (*K*, *τ*_*P*_) along such isocline gives an optimal fit to the experimentally observed phase difference as illustrated in Fig 2D.

**Fig 3:**
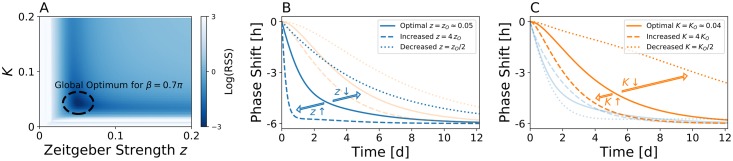
Constraining *Zeitgeber* and coupling parameters by jet-lag data. Given any *β* that allows for a reproduction of the experimentally observed phase difference between Per1 and Bmal1 oscillations under constant light conditions, the parameter (*z*) that determines the *Zeitgeber* strength can be estimated from experimental jet-lag data as demonstrated here for *β* = 0.7*π*. A) Fitness landscape in the parameter plane of coupling constant *K* and *Zeitgeber* strength *z*. Please note that for each *K*, we assigned the parameter *ω*_*P*_ (and thus also *ω*_*R*_) along the iscoline of Fig 2C such that the experimentally observed phase difference between Per and Bmal-Rev loops is reproduced. Colors denote the logarithm of the residual sum of squares (RSS) between the simulated and experimental jet-lag dynamics as depicted in Fig 2E. B,C) Impact of *Zeitgeber* intensity (*z*, panel B) and coupling strength *K* between intracellular feedback loops (panel C) on jet-lag behavior of the Per (blue lines) and Bmal-Rev (orange lines) dynamics.

The sets of parameters that optimally fit *Per1* and *Bmal1* gene expression rhythms under free-running conditions can be further constrained by additionally considering the entrainment to light cycles. We therefore quantitatively compare the simulated response to a 6h advancing phase-shift in the light schedule (jet-lag) with the corresponding experimentally obtained mRNA profiles from [20], see Fig 2E. By calculating the residual sum of squares (RSS) between simulated and experimental time series for different combinations of coupling strength *K* and *Zeitgeber* intensities *z*, we can identify for any given *β* a global optimum in the corresponding fitness landscape, see Fig 3A. In case of *β* = 0.7*π*, such global optimum can be found for *τ*_*P*_ ≈ 24.38h, *τ*_*R*_ ≈ 24.68h, *K* ≈ 0.043 and *z* ≈ 0.051, compare Fig 3A. A sensitivity analysis that considers changes in the *Zeitgeber* intensity *z* and coupling strength *K* reveals that *Zeitgeber* intensity *z* mainly determines the time scale of Per response to jet-lag, while coupling strength *K* mainly determines to which extent response dynamics of Bmal1 lags behind that of Per, compare Fig 3B and 3C and S5A and S5B Fig. Generally, a larger (smaller) *Zeitgeber* intensity *z* or coupling strength *K* accelerates (decelerates) the corresponding response dynamics.

As discussed above, we considered symmetrical couplings between the Per and Bmal-Rev loops (i.e. *K*_*P*_ = *K*_*R*_ in Fig 2B) mainly for the sake of simplicity. Asymmetries in the coupling topology may additionally contribute to transient dissociation dynamics. Given a constant overall coupling *K* = *K*_*R*_ + *K*_*P*_ with *K*_*R*_ = *pK*, *K*_*P*_ = (1 − *p*)*K* and *p* ∈ [0, 1], a stronger impact of the Per loop on Bmal-Rev loop (*p* > 0.5) in comparison to the opposite situation generally leads to a longer transient dynamics of the Bmal-Rev compared to the Per loop after a 6h phase advancing jet-lag, see S5C Fig.

**Fig 4:**
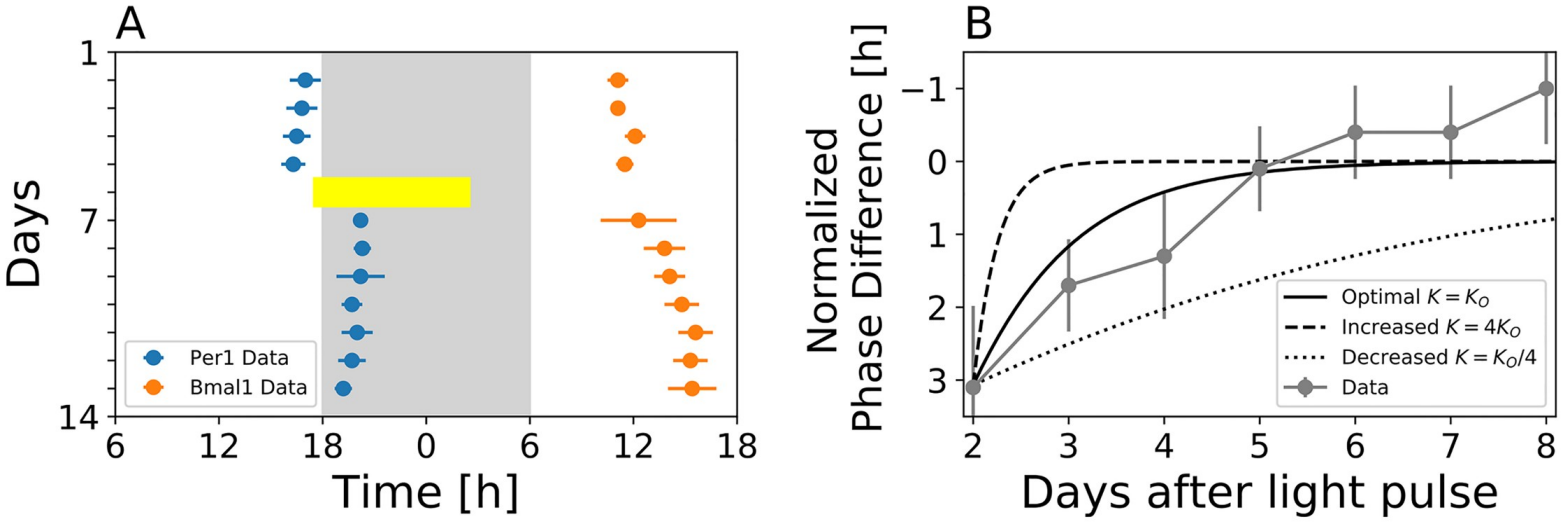
Differential response after light pulse applications depends on the coupling strength between the Per and Bmal-Rev feedback loops. A) Mean acrophases and the corresponding standard deviation of *Per1-luc* (blue) and *Bmal1-ELuc* (orange) oscillations (*n* = 3 for each reporter construct), recorded *in vivo* from single-transgenic adult mice as described in [21]. At day 5, a 9h light pulse was applied at CT11.5h (yellow bar). B) Simulated dynamics of the phase difference (Δ*θ*(*t*)) between Per and Bmal-Rev loops as given by Eq (3) of Section *Materials and Methods* for different coupling strength *K* (black lines) in comparison with the corresponding experimental data (gray). Phase differences have been normalized such that the phase difference between Per and Bmal1 oscillations one day prior to the application of the light pulse is set to zero in both, simulated and experimental time courses. Application of the light pulse leads to a perturbation from the Per1-Bmal1 free-running phase difference by approximately 3h that subsequently re-adapts within 5 days. Note that panel A is a modified reproduction of Figure 1 C in [21].

After application of a 9h light pulse at CT11.5h to adult mice, differential responses of *Per1-luc* and *Bmal1-ELuc* expression rhythms have been observed under *in vivo* recordings, see Fig 4A and reference [21]. The experimentally observed phase dynamics that, after initial acute *Per1-luc* response to light, converges towards the steady phase difference Δ*θ*^⋆^ in the long run, can be reproduced by our conceptual model, using the “optimal” parameter set, *i.e.*, the parameter set that optimally reproduces the above described free-running and jet-lag data (for *β* = 0.7*π* as highlighted in Fig 3A), see Fig 4B. Again a larger (smaller) coupling strength *K* would lead to faster (slower) recovery of the steady state phase difference Δ*θ*^⋆^, compare the dashed (dotted) line in Fig 4B.

In conclusion, our conceptual model that assumes interlocking of oscillating Per and Bmal-Rev intracellular feedback loops, where only the Per loop receives direct light input, successfully describes experimental *Per1* and *Bmal1* mRNA oscillations under free-running conditions as well as dissociating dynamics upon jet-lag and 9h light pulses, in case of weak enough coupling between both loops.

We examine the robustness of our results by exploiting a slightly more complex conceptual model that additionally considers amplitude effects in a system of two (mean-field) coupled Poincaré oscillators, representing again the Per and Bmal-Rev loops. Within this model, the results obtained from the phase model can be robustly reproduced, see S6 Fig. Interestingly, models that assume either a self-sustained or a slightly damped Bmal-Rev oscillator are able to reproduce the experimental data. Coupling between the Per and the Bmal-Rev loop needs to be strong enough to allow for synchronized free-running oscillations but weak enough to allow for dissociating dynamics after perturbations of the system, compare S6(A)-S6(E) and S6(F)-S6(J) Fig. However, self-sustained oscillations of the Bmal-Rev loop facilitate dissociating dynamics as indicated by a larger set of parameters that lead to a good fit to experimentally observed jet-lag dynamics, compare S6(C) and S6(H) Fig.

### A minimal three-gene molecular circuit model of interlocked feedback loops successfully recapitulates free-running and light-response behavior

Can the results from our conceptual modeling be reproduced by contextual molecular circuit models that describe the network of transcriptional regulations between the core clock genes? It has been shown that condensed molecular circuit models, accounting for the interplay of cis-regulatory elements while transforming post-transcriptional regulations (*e.g.*, phosphorylation, nuclear transport, complex formation) into explicit delays, are able to faithfully reproduce experimentally observed periods and phases under free-running conditions [31]. Using a five gene model of the mammalian core oscillator network—consisting of the Bmal, Dbp, Rev, Per and Cry genes—Pett and colleagues showed that sub-networks of this model are enough to generate essential properties of circadian oscillations, while the full set of the five gene network is not needed for this purpose [39]. Such sub-modules include the auto-inhibitory regulation of *Per* and *Cry* gene expression, the Bmal-Rev loop as well as a Per-Cry-Rev repressilator motif, among others. By fitting the five gene model to clock gene expression data from 10 different tissues, it has been shown that the relative importance and balance between the sub-loops differ in a tissue-specific manner [40]. Here we aim to find a minimal molecular circuit model that accounts for the experimentally observed dynamics under free running conditions as well as dissociating dynamics of clock genes perturbed by the light input (jet-lag, light pulses).

**Fig 5:**
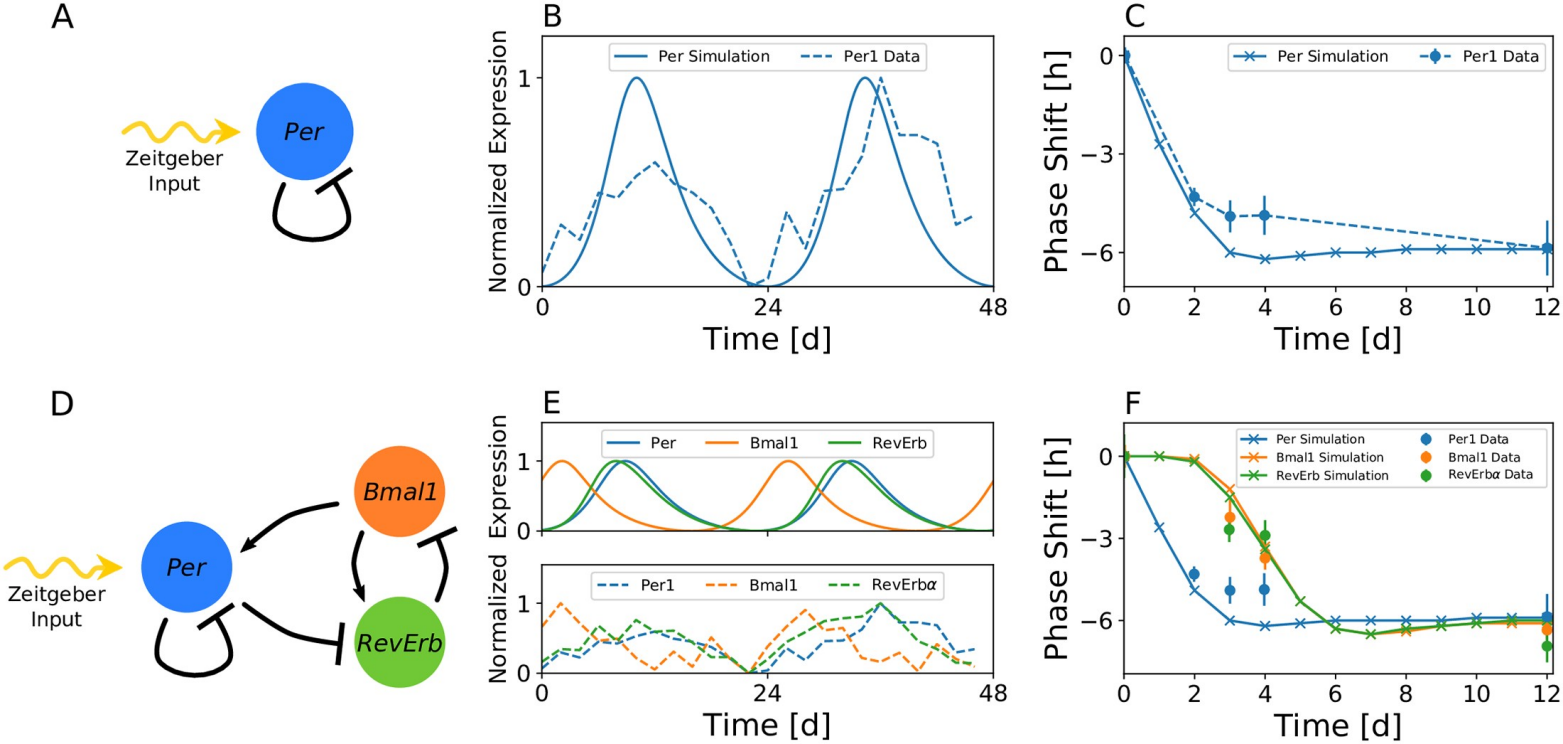
Free-running and differential jet-lag responses can be reproduced by a three-gene molecular circuit model. A) Network structure of the auto-inhibitory Per1 loop driven by light (*Zeitgeber* signal). B) The single auto-inhibitory Per1 loop is sufficient to reproduce experimentally observed Per1 gene oscillations under free running conditions for suitable sets of parameters. Simulated Per1 dynamics as well as the corresponding experimental time series from Zhang *et al*. [33] are depicted by bold and dashed lines, respectively. C) For an appropriate *Zeitgeber* intensity (*z* = 0.21), the experimentally observed Per1 mRNA response to a 6h phase advancing jet-lag can be reproduced by the light-driven single auto-inhibitory Per1 loop. D) Network structure of the light driven auto-inhibitory Per1 loop, interlocked with the Bmal-Rev loop. E) A three variable model, consisting of Per1, Bmal1 and Rev-Erb*α* genes and their transcriptional regulatory interactions is able to reproduce experimentally observed Per1, Bmal1 and Rev-Erb*α* gene expressions under free running conditions for suitable sets of parameters. F) For a suitable, intermediate strength of coupling between the Per and Bmal-Rev loops, *i.e.* *c*_*R*_ = 35, the experimentally observed differential response of Per1, Bmal1, and Rev-Erb*α* genes to a 6h phase advancing jet-lag can be observed. The term “intermediate” denotes strong enough coupling to synchronize the Per and Bmal-Rev loops but weak enough coupling to allow for a dissociation between them.

It is known from theoretical studies that a single delayed negative feedback loop is sufficient to exhibit oscillations [31, 41–43], see Fig 5A for a schematic drawing of the corresponding network motif. By incorporating intermediate regulatory steps—such as translation, post-transcriptional or post-translational modifications—along this loop into explicit delays, we can model such single negative feedback loop by a one-variable, four parameter delay differential equation, see Eq (10) in *Materials and Methods*. For a suitable set of parameters, such one-variable model, based on the auto-inhibition of Per gene expression, is capable of reproducing experimentally observed *Per1* gene expression rhythms under free-running conditions, see Fig 5B. When driven by a *Zeitgeber* signal of appropriate strength (*z* = 0.21), we can mimic the response of *Per1* gene expression rhythms to a 6h phase advancing jet-lag, see Fig 5C. Similar to the conceptual model described in the previous subsection, we incorporate the impact of light as a *Zeitgeber* signal by an additive (activatory) effect upon *Per1* transcription. Although our model was not optimized for this sake, experimentally observed entrainment phases of *Per1* mRNA [44] around midday can be reproduced by a *Zeitgeber* intensity of *z* = 0.21 and model parameters as described in Section *Materials and Methods*, compare S7A Fig. Since the Per-one-loop model only describes the dynamics of a single clock gene, it is insufficient to reproduce the transiently dissociating dynamics of clock gene expression after perturbations of the system.

**Fig 6:**
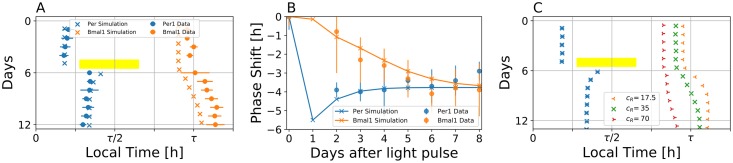
A three-gene molecular circuit model accounts for experimentally observed differential dynamics induced by a 9h light pulse. A) Simulated (crosses) and experimental (circles) acrophases of Per1 and Bmal1 gene oscillations, subject to a 9h light pulse. The yellow bar denotes the 9h light pulse in the simulated dynamics. A *Zeitgeber* intensity of *z* = 0.45 was used during the 9h light pulse. In analogy to the corresponding experimental conditions, the light pulse was applied 2.3h after the peak of Per1 expression. Note that experimental acrophase data (circles) are the same as those in Fig 4A. The time scale represented on the x-axis has been normalized to account for different free-running periods in the light pulse experiment and the three-gene model fitted to the high throughput data. B) Dynamical evolution of the simulated (bold lines) and experimentally observed (dashed lines) phase shift of Per1 (blue) and Bmal1 (orange) genes induced by a 9h light pulse, as depicted in panel (A). C) Simulated acrophases of Per1 (blue) and Bmal1 (orange, green, red) genes, subject to a 9h light pulse, for different parameter values *c*_*R*_. Compared to the case of *c*_*R*_ = 35 depicted in panel (A), a smaller value of *c*_*R*_ = 17.5 leads to a faster response of Bmal1 to the light pulse, while a larger value of *c*_*R*_ = 70 leads to a slower response. Different values of *c*_*R*_ are highlighted by different marker symbols.

We therefore expand the single auto-inhibtory Per-one-loop model by interlocking it with the two-gene Bmal-Rev composite negative feedback loop, see Fig 5D for a schematic drawing of the corresponding network motif. For a suitable set of parameters, such three-gene model is able to reproduce the experimentally observed gene expressions under free running conditions, see Fig 5E. The extended model of interlocked Per and Bmal-Rev negative feedback loops is able to mimic the experimentally observed faster re-entrainment of *Per1* gene compared to *Bmal1* and *RevErbα* genes after a 6h phase advancing jet-lag, see Fig 5F. As depicted in Fig 6A and 6B and S8 Fig, a relatively faster response of simulated *Per* dynamics to a 9h light pulse, applied approximately 2h after the *Per* oscillation peak under DD free-running conditions, can be observed when compared to the corresponding Bmal1 response. For a suitable set of parameters, simulated time scales of transient dynamics are in good agreement with corresponding experiment (Fig 6A and 6B). As discussed in the conceptual model, response times of simulated *Bmal1* gene expression with respect to perturbations in the light schedule depend on the “coupling strength” between Per and Bmal-Rev loops. Here, the “coupling strength” can be associated with parameter *c*_*R*_ that affects the strength of inhibition of RevErb transcription by increasing the expression levels of Per gene. A smaller value of *c*_*R*_ (*i.e.*, increasing “coupling strength”) leads to a faster response of Bmal1 to a 9h light pulse, while a larger value of *c*_*R*_ (*i.e.*, decreasing “coupling strength”) slows the Bmal1 response in comparison with the nominal value of *c*_*R*_ = 35, see Fig 6C and S9A and S9C Fig for a corresponding jet-lag analysis. These differential response times can translate into differential (instantaneous) periods of Bmal1 and Per dynamics immediately after the perturbations, see S9B and S9D Fig. Depending on the type and phase of perturbation, as well as the variable, on which the perturbation acts, transiently dissociating dynamics ranging from a couple of days up to several weeks can be observed, compare S9 and S10 Figs.

As the coupling between Per and Bmal-Rev feedback loops is further weakened, qualitatively different transient dynamics may emerge, in which the phase of Bmal1 moves towards a phase-advancing direction and subsequently crosses the phase of Per, see S9A Fig. The latter situation may analogously take place when Per and Bmal-Rev feedback loops are completely desynchronized, with the exception that no stable phase locking emerges after transient dynamics decayed. Thus, a complete dissociation can only be experimentally distinguished from long transient dissociating dynamics for recordings on a sufficiently long time interval.

## Discussion

Per1/2 and Bmal1 reporters have been routinely used in various circadian studies and across different tissues, where slight differences in period between the two reporters have been known. However, it has been only recently realized that these differences can be systematic and are dependent upon specific external perturbations applied to the clock system. Abrupt alterations in the *Zeitgeber* signal such as jet-lags and disruptive light pulses can lead to a dissociation of Per and Bmal1 gene expression oscillations in the SCN of live animals [19–21]. A similar dissociation has been observed after preparation of SCN slices, *i.e.*, after transferring the clock system from *in vivo* to *in vitro* conditions [21, 22]. Furthermore, subsequent culture medium exchanges elicit differential phase-dependent phase shifts of *Per2* and *Bmal1* oscillations [23]. These data have been recorded at the SCN tissue level. The question remains whether the observed dynamical dissociation occurs at the single cell level or between disjoint subsets of the SCN neurons. Our surrogate data analysis, based on a comparison between experimentally obtained bioluminescence recordings and the corresponding *in silico* generated data, favors the assumption that the dynamical dissociation occurs at the single cell level. It should be noted, however, that the surrogate data of both hypotheses can show qualitatively similar features in case that the cell densities are high or the cell sizes are large that signals of neighboring cells overlap in individual pixels, see S2 Fig. Although our analysis is based on reasonable assumptions on the cell density, a definitive answer to the single cellular dissociation may require experiment on simultaneous measurements of *Per* and *Bmal1* gene expressions in isolated or sufficiently dispersed cells.

Ubiquity of circadian rhythms in broad biological processes and organisms suggests that, despite the common underlying mechanism of negative feedback loop, there can be diverse biological implementations [45]. In mammals, more than a dozen clock genes have been described to constitute the core clock network, including *Per*, *Cry*, *Bmal*, *RevErb* and *Ror* genes [46]. These genes form multiple feedback loops that have different effects on regulating their own expression. Functionally redundant loops ensure robustness, while heterogeneous combinations of negative and positive feedback loops can provide higher flexibility in oscillations than a single feedback loop, such as a broader range of tunability [47]. Similarly, heterogeneous interaction of clocks may have a wider encoding capability in the tissue-level network [48], which can be reduced to two-oscillator dynamics [49]. To elucidate the transient dissociation of clock genes, we have divided the molecular feedback loops into a Per and a Bmal-Rev loop. By means of conceptual and detailed mechanistic molecular circuit models, we could show that such dissociation at the single cell level is indeed plausible within a system of multiple interlocked feedback loops.

The conceptual phase oscillator model highlights design principles for the dissociating clock gene dynamics. Oscillation phases under free-running conditions as well as time scales of transient dissociation between the Per and Bmal-Rev loops after perturbations of the system can be realized by well balanced period differences and coupling strength between the two loops. Responses of the Per loop to perturbations in the light schedule largely depend on the effective *Zeitgeber* strength. Time scales of the transient dissociation between Per and Bmal-Rev loops, which are long enough to be analogous to “internal desynchronization,” are determined mainly by their coupling strength and their individual periods.

Due to the abstract nature and generality of the phase oscillator model, which is solely based on two coupled oscillators with one entity (the Per loop) being unilaterally driven by light, the results can be transferred to interpret analogous situations in other experimental settings. Based on neurotransmitter and neuropeptide release as well as afferent and efferent connections, the SCN has been functionally divided into different sub-regions, *e.g.*, core and shell [49, 50]. Neurons in the SCN core release vasoactive intestinal polypeptide (VIP) and gastrin releasing peptide (GRP) and receive most of the afferent inputs via the retinohypothalamic tract that mediates light information to the SCN. This core region is surrounded by the shell region that mainly releases arginine vasopressin (AVP) and is less dense in afferent synaptic inputs mediating photic cues. Analogously to the *Per1*-*Bmal1* dissociation in response to a 6 h advance of *Zeitgeber* cycles, neurons in the SCN core entrain faster to the phase shifts compared to those in the SCN shell [51]. Here, the core that receives photic input is functionally similar to the Per loop, while the shell corresponds to the Bmal-Rev loop. Intermediate coupling between the core and the shell, that is strong enough to allow their synchronized oscillations but weak enough to exhibit “internal desynchronization” in the process of adjustment to the new phase, may explain the data.

As a more realistic molecular circuit model, a minimalistic three-gene network has been further proposed, in which the negative auto-regulatory Per loop is interlocked with the Bmal-Rev composite negative feedback loop. Regulatory interactions between the three genes have been inferred from experimentally validated interactions via cis regulatory E-box, D-box and ROR elements as proposed in [31, 39, 40]. An intermediate coupling strength between the two loops, that is strong enough to exhibit synchronized oscillations but weak enough to allow for transient differential dynamics of the two, can recapitulate experimentally observed dissociating dynamics induced by jet-lags and light pulses. Depending on inter-loop coupling, the dissociation may last from a couple of days up to several weeks (compare Figs 5 and 6, S9 and S10 Figs). Previously published molecular circuit models of the mammalian circadian clock mainly focused on steady state free-running, entrainment to *Zeitgeber* cycles, and mutant behaviors [5, 14, 31, 39, 40, 52–54]. We here provide a mammalian intracellular clock model that additionally accounts for the transient differential behavior of clock genes in response to light perturbations. We highlight that even a minimalistic gene regulatory network, composed of not more than three genes, is able to explain a variety of complex data sets.

The minimalistic three-gene network, composed of *Bmal1*, *Per*, and *RevErb* genes, represents only a subset of known mutual clock gene regulations. This structure can be interpreted as a sub-module, or motif, that embeds into a more complex network of clock gene regulations, including additional elements such as *Cry*, *Ror* and *Dec* genes. We therefore tested whether transient dissociating dynamics of the clock genes can be identified even in a larger core clock model as described in [39, 40]. Therein, the network of 20 known clock genes has been condensed into gene regulatory interactions of five groups of genes (see S11A Fig and [39, 40]). After re-analyzing and optimizing the solutions from [39], which were obtained by fitting to SCN-specific data sets with additional “sub-network conditions” (see S1 Text), we found that dissociation of *Bmal1* and *Per* oscillations is possible even within this densely connected model. This implies that the present results on dissociating dynamics of clock genes are quite general and do not depend on the complexity of intracellular gene regulatory networks.

Time scales of transiently dissociating dynamics, ranging from couple of days to several weeks, depend on intra-loop coupling strengths, perturbation phases and the clock component(s), on which a given perturbation acts (S10 Fig). Depending on the tissue, different clock components have been shown to receive signals from different signaling pathways. Photic stimuli activate Per expression in SCN neurons of the brain [34], while glucocorticoids specifically activate expression of Per1 in human and mouse peripheral tissues [55]. Adenosine monophosphate-activated protein kinase (AMPK) phosphorylates and thereby destabilizes CRY1 in peripheral clocks [56, 57]. Peroxisome proliferator-activated receptor (PPAR) transcription factors regulate expression of RevErb*α* in human hepatocytes [58]. Additionally, pharmacological compounds such as, e.g., the CK1*δ* inhibitor PF-670462 can perturb specific clock components in a dose-specific manner [59, 60]. It will be interesting to test if and to which extend such specific perturbations of certain clock gene expressions in peripheral tissues lead to transient dissociation dynamics. A theoretical analysis of differential response dynamics as proposed in this manuscript may help to untangle the balances of intra-cellular feedback loop regulations in peripheral clocks.

The working hypothesis of autonomously oscillating, yet coupled, intra-cellular feedback loops has a long tradition in chronobiology. In the late 1970s, long before molecular key players of mammalian circadian rhythm generation have been identified, Pittendrigh, Daan and Berde proposed two separate coupled oscillators as a means to explain splitting of behavioral activity under constant light (LL) in *Mesocricetus auratus* [61, 62]. They have been termed as morning (M) and evening (E) oscillators with respect to the timing of the corresponding activity components before the splitting. Throughout the last decades, this dual oscillator concept has been applied to interpret different kinds of circadian phenomena, including bimodal activity patterns, photoperiodic entrainment properties, after-effects and internal desynchronization [63]. In mammals, different candidate genes have been proposed to constitute such dual morning-evening oscillator system, although direct evidence for the existence of intracellular M and E oscillators is still lacking. Based on differences in free-running oscillation phases and light responses, Daan *et al*. hypothesized that *Per1* and *Cry1* may constitute a M oscillator, while *Per2* and *Cry2* act as an E oscillator within a single cell [64], a concept that was later studied computationally [65]. Nuesslein-Hildesheim *et al*. proposed a dual oscillator system as composed of light-sensitive *mPer* and light-insensitive *mCry* cycles [66]. Our model provides an alternative dual-oscillator perspective based on the light-sensitive Per negative feedback loop, interlocked with the light-insensitive Bmal-Rev feedback loop. It has been shown in [21] that phase shifting behaviors of *Per1* and *Bmal1* resemble those of the activity onset and offset in behavioral rhythms, respectively. This suggests that Per and Bmal-Rev feedback loops may explain the behaviors associated with M and E oscillators.

Circadian clocks serve as an internal reference of time for activity on-set and off-set locked to the phases of day and night. While the period of day-night cycles remains fixed, the day-length varies across the seasons. Phases of the activity on-set and off-set also change through the seasons. The biological clock should, therefore, be not just a robust timekeeper of 24 h cycle but also a flexible clock that adapts to such varying photoperiods. Differential responses to light between M and E oscillators can lead to a photoperiod-dependent adjustment of their phase difference, which can ultimately explain seasonal changes in behavioral activity onset, offset and activity duration (*α*) [48, 63]. Our model suggests that such flexible maintenance of time (or “dynamical plasticity” as recently discussed for the plant circadian clock [67]) is possible within a single cell. Since seasons affect all species on the planet, it would be interesting to investigate whether transient dissociations of clock genes can be analogously observed in other non-mammalian organisms such as plants, flies, or even unicellular organisms.

## Materials and methods

### Experimental data

#### Bioluminescence recordings

The bioluminescence recordings of transgenic mice have been obtained from reference [21]. The data from SCN slice preparations, as shown and analyzed in Fig 1, has been obtained from *in vitro* brain slice preparations of neonatal transgenic mice, expressing a *Bmal1-ELuc* and *Per1-luc* reporter construct at the same time. By using a filter wheel setup with an exposure time of 29min under each condition (*i.e.*, with and without filter), both signals have been separated such that a sampling interval of Δ*t* = 1h results for the time series of *Bmal1-ELuc* and *Per1-luc* gene expression. Acrophases of *Bmal1-ELuc* and *Per1-luc* reporter constructs before and after a 9h phase light pulse, as shown in Figs 4 and 6, have been directly taken from [21]. The behavioural data have been obtained from *in vivo* optical-fiber recordings of the SCN in single transgenic freely moving adult mice, expressing either a *Bmal1-ELuc* or a *Per1-luc* reporter construct. Further details on the experimental protocols can be found in [21].

#### Jet-lag experiments

In [20], changes in the rhythmicity of SCN clock gene expression after a 6h phase advance, following equinoctial LD12:12 entrainment, has been examined by *in situ* hybridization. SCN tissue has been hybridized with labeled anti-sense RNA a day before and at days 2, 3, 4, and 12 after the 6h phase advance at 6 time points per day at an equidistant sampling interval of 4h. A sine fit to the time dependent RNA profiles, as determined by densitometry, quantifies the phase shifting dynamics induced by the 6h phase advance. Such original phase shift data of Figure 2 from [20] has been extracted by the free online software WebPlotDigitizer [68] and further used to constrain our model parameters with respect to entrainment dynamics, see Figs 2E and 5F.

### Surrogate data

Statistical hypothesis testing was applied to the bioluminescence recordings of SCN slice, expressing both *Bmal1-ELuc* and *Per1-luc*, by the method of surrogate [25]. The surrogate *in silico* data that mimics experimentally obtained time-lapse recordings of double-luciferase bioluminescence reporter constructs has been created based on two null hypotheses. The first null hypothesis $H_{0}^{\left(1\right)}$ states that each of *N* single cells produces both Bmal1 and Per1 signals that differ in period, *i.e.*, Bmal1 and Per1 are assumed to be dissociated at the single cell level. The second null hypothesis $H_{0}^{\left(2\right)}$ assumes that half of *N* single cells produce only a Bmal1 signal, while the other half of single cells produce only a Per1 signal with a period different from the one of the Bmal1 signal. In accordance with the experimental protocol that uses a filter wheel to seperate signals of different wavelength from *Bmal1-ELuc* and *Per1-luc* reporters, we construct a stack of two signals, the Bmal1 and Per1 signal. Spillover effects are neglected. Details of the surrogate data generation procedure are as follows: First, we locate positions of *N* single cells randomly from a two-dimensional uniform distribution. Second, periods for Bmal1 and/or Per1 signal are assigned to each of the the *N* cells based on the null hypothesis $H_{0}^{\left(1\right)}$ or $H_{0}^{\left(2\right)}$. Third, Bmal1 and/or Per1 signals are generated for each cell for a total duration of 0d < *t* < 12d at a sampling rate of Δ*t* = 1h, following the experimental protocol of [21]. For the sake of simplicity, we assume that the signal *s*_*i*_(*t*), either *Bmal1-ELuc* or *Per1-luc*, in single cell *i* is described by a cosine function of maximal intensity *I*_*i*_, initial oscillation phase *ϕ*_*i*_, and period *τ*_*i*_, *i.e.*, $s_{i}(t)≔\frac{I_{i}}{2}(1+\text{cos}(\frac{2\;\pi }{\tau_{i}}t+\phi_{i}))$. Fourth, single cell intensities of Bmal1 and Per1 signals that have been calculated at discrete positions are convoluted with a two-dimensional *Gaussian* kernel of standard deviation *σ*_*G*_ that resembles the experimentally observed size of a neuron. Subsequently, all convoluted signals are superimposed and intensity values are calculated for discrete grid positions such that the resulting grid resembles dimension of the original experimental image as well as resolution of the camera, *i.e.*, diameter of the *in silico* SCN neuron has the same dimension in units of pixels as in the corresponding experiments. Since SCN slice preparations are three-dimensional objects with neurons distributed along all three spatial dimensions, we assume *M* = 3 layers of neurons in our surrogate time lapse imaging, *i.e.*, steps 1-5 are repeated for each of the *M* layers and the signals are superimposed by assuming that the intensity drops by 50% at each layer to mimic reflection and absorption processes. Finally, observational *Gaussian* noise of zero mean and standard deviation *σ*_*n*_ is added to each grid element at each time point independently. Step-wise procedures to generate the surrogate data are illustrated in S1 Fig.

### Time series analysis

Experimental data for *Per1-luc* and *Bmal1-ELuc* bioluminescence recordings of SCN slices as well as the corresponding surrogate time lapse movies are analyzed using the same custom written Python script. Time series from the experimental data are baseline detrended by means of a Hodrick Prescott filter [22], using the hpfilter function of the statsmodels Python module for a smoothing parameter $\lambda =0.05{\left(\frac{24\text{h}}{\Delta t}\right)}^{4}$ with Δ*t* being the sampling interval, as described in [69]. Oscillation periods of the detrended signals are further analyzed by a Lomb Scargle periodogram [70] in the period range of [4*h*, 48*h*] using the lombscargle function from the signal module of the Scientific Python package.

Additionally to the Lomb Scargle periodogram, a simple harmonic function

$$\begin{array}{c} y_{i}\left(t\right)=(a_{i}\cos(\frac{2\pi }{\tau_{i}}t)+b_{i}\sin(\frac{2\pi }{\tau_{i}}t)) \end{array}$$

fit has been applied to the detrended time series in order to estimate the oscillatory parameters. Beside oscillation periods *τ*_*i*_, amplitudes and phases can be determined as $A_{i}=\sqrt{a_{i}^{2}+b_{i}^{2}}$ and *ϕ*_*i*_ = arctan 2(*b*_*i*_, *a*_*i*_), respectively.

### Conceptual phase oscillator model

As a conceptual model of intracellular circadian oscillation, two coupled phase oscillators [71] are constructed as follows (Fig 2B),

$$\begin{array}{cc} {\dot{\theta }}_{P} & =\omega_{P}+K_{R}\sin\left(\theta_{R}-\theta_{P}-\beta \right)+\underset{Z(t)}{\underset{︸}{z\sin(\frac{2\;\pi }{T}t-\theta_{P}+\phi_{0})}}, \end{array}$$ (1)

$$\begin{array}{cc} {\dot{\theta }}_{R} & =\omega_{R}+K_{P}\sin\left(\theta_{P}-\theta_{R}+\beta \right). \end{array}$$ (2)

*θ*_*P*_ and *θ*_*R*_ represent oscillation phases of the Per and Bmal-Rev loops, respectively. $\tau_{P}≔\frac{2\;\pi }{\omega_{P}}$, $\tau_{B}≔\frac{2\;\pi }{\omega_{B}}$ and *T* denote intrinsic periods of the Per loop, Bmal-Rev loop, and the *Zeitgeber* signal, respectively. *K*_*R*_ and *K*_*P*_ determine strength of interaction between the Per and Bmal-Rev loops, while *z* denotes strength of the light input. Parameter *β* allows for a flexible adjustment of the steady state phase difference Δ*θ* ≔ *θ*_*P*_ − *θ*_*R*_ in the limit of vanishing frequency differences (Δ*ω* ≔ *ω*_*P*_ − *ω*_*R*_ = 0) or infinite coupling strength (*K*_*P*_, *K*_*P*_ → ∞ for finite Δ*ω*).

Under free-running conditions (*i.e.*, *z* = 0), Eqs (1) and (2) can be rewritten as

$$\begin{array}{c} \frac{\text{d}\Delta \theta }{\text{d}t}=\Delta \omega -\left(K_{P}+K_{R}\right)\sin\left(\Delta \theta +\beta \right)\;. \end{array}$$ (3)

Synchronization (*i.e.*, phase-locking) between both loops, given by condition $\frac{\text{d}\Delta \theta }{\text{d}t}=0$, occurs for all sets of parameter that fulfill the inequality

$$\begin{array}{c} |\frac{\omega_{P}-\omega_{R}}{K_{P}+K_{R}}|<1\;. \end{array}$$ (4)

In case of such overcritical coupling or synchronization, both loops oscillate with a common angular velocity as given by the weighted arithmetic mean

$$\begin{array}{c} \omega^{⋆}=\frac{K_{P}\;\omega_{P}+K_{R}\;\omega_{R}}{K_{P}+K_{R}} \end{array}$$ (5)

of their individual frequencies and a stable phase relationship

$$\begin{array}{c} \Delta \theta^{⋆}=\arcsin(\frac{\omega_{P}-\omega_{R}}{K_{P}+K_{R}})-\beta \;. \end{array}$$ (6)

Here, the phase difference $\Delta \theta^{⋆}\in [-\beta -\frac{\pi }{2},-\beta +\frac{\pi }{2}]$ between *θ*_*P*_ and *θ*_*P*_ solely depends on *β*, the sum of the coupling strength *K*_∑_ = *K*_*P*_ + *K*_*R*_ and the frequency difference Δ*ω* = *ω*_*P*_ − *ω*_*R*_.

In case of symmetric coupling (*K*_*P*_ = *K*_*R*_ ≕ *K*), Eq (5) is simplified to

$$\begin{array}{c} \omega^{⋆}=\frac{\omega_{P}+\omega_{R}}{2} \end{array}$$ (7)

and Eq (6) can be rewritten as

$$\begin{array}{c} \Delta \theta^{⋆}=\arcsin(\frac{\omega^{⋆}-\omega_{R}}{K})-\beta =\arcsin(\frac{\omega_{P}-\omega^{⋆}}{K})-\beta \;. \end{array}$$ (8)

### Conceptual amplitude-phase model

In order to consider amplitude effects, we introduce a conceptual model based on two coupled Poincaré oscillators. For the sake of simplicity, we assume that both oscillators couple symmetrically by means of a mean field similar to models discussed in [72]. The corresponding equations in their complex form read as

$$\begin{array}{c} \frac{\text{d}z_{j}}{\text{d}t}=(\lambda_{j}\;\left(A_{j}-r_{j}\right)+i\;\frac{2\pi }{\tau_{j}})z_{j}+K\;e^{i\phi }\;\sum_{j=P,R}z_{j} \end{array}$$ (9)

where $z_{j}\in \mathbb{C}$ with *j* ∈ {*P*, *R*} are the complex variables describing the oscillations of the Per (*j* = *P*) and Bmal-Rev (*j* = *R*) loop, respectively. λ_*j*_ denote radial relaxation rates, *A*_*j*_ individual amplitudes, *τ*_*j*_ intrinsic periods, *K* the coupling strength, *ϕ* the coupling phase and *i* the complex element. By setting *A*_*j*_ = 0, one can transform the self-sustained limit cycle oscillator into a damped one.

As in [38], we model the effect of a given *Zeitgeber* on the Per loop by adding the *Zeitgeber* function $Z(t)=z\;\text{sin}(\frac{2\pi }{T}t+\phi_{0})$ to differential Eq $\frac{\text{d}x_{P}}{\text{d}t}$, describing the x-variable of $\frac{\text{d}z_{P}}{\text{d}t}$ in Cartesian coordinates.

### Detailed mechanistic model

Contextual molecular circuit models are developed, based on the interplay of E-box, D-Box and RRE cis-regulatory elements, as previously published [31, 39, 40]. While transcriptional activation and repression are described by means of (modified) Hill functions, degradation is modeled via first order kinetics. Instead of implicit delays implemented in large reaction chains, translation as well as post-transcriptional and post-translational modifications are condensed into explicit delays.

Using a previously published model [31] of Per gene expression dynamics

$$\begin{array}{c} \frac{\text{d}P\left(t\right)}{\text{d}t}={\left(\frac{v_{P}}{k_{P}+P\left(t-T_{P}\right)}\right)}^{2}-d_{P}P\left(t\right)+Z\left(t\right) \end{array}$$ (10)

and the (modified) corresponding parameter set (*v*_*P*_ = 1, *k*_*P*_ = 0.1, *d*_*P*_ = 0.25 h^−1^, $T_{P}=8.333\;\text{h}$), we demonstrate in Fig 5A and 5B that a single negative feedback loop is able to generate circadian oscillations.

In order to mimic dissociating dynamics between the Per and Bmal-Rev feedback loops, the Per single gene model of Eq (10) has been interlocked with a two-variable model, describing the Bmal-Rev negative feedback loop. The full set of Eqs read as

$$\frac{\text{d}P\left(t\right)}{\text{d}t}\;=\;{\left(\frac{v_{P}}{k_{P}+P\left(t-T_{P}\right)}\right)}^{2}{\left(\frac{c_{P}+b_{P}B\left(t-T_{B}\right)}{c_{P}+B\left(t-T_{B}\right)}\right)}^{2}-d_{P}P\left(t\right)+Z\left(t\right),$$ (11)

$$\frac{\text{d}B\left(t\right)}{\text{d}t}\;=\;{\left(\frac{v_{B}}{k_{B}+R\left(t-T_{R}\right)}\right)}^{2}-d_{B}B\left(t\right),$$ (12)

$$\frac{\text{d}R\left(t\right)}{\text{d}t}\;=\;{\left(\frac{v_{R}+b_{R}B\left(t-T_{B}\right)}{k_{R}+B\left(t-T_{B}\right)}\right)}^{3}{\left(\frac{c_{R}}{P\left(t-T_{P}\right)+c_{R}}\right)}^{3}-d_{R}R\left(t\right).$$ (13)

Under the assumption that light acutely induce Per transcription, time-dependent *Zeitgeber* function *Z*(*t*) appears as an additive term in Eqs (10) and (11) and a square wave signal of period *T* and intensity *z* is implemented as described previously [38]. In comparison to the “full” five or six gene models of [31, 39, 40] that additionally include *Cry1*, *Ror*, and *Dbp* clock genes, here we considered only those genes and regulatory interactions that are necessary and sufficient for the occurrence of free running oscillations and entrainability of both Per and Bmal1 genes to the *Zeitgeber*. Along these lines, RevErb is a necessary network node, since the inhibitory effect of Per protein on RevErb transcription is transmitted towards Bmal1 via the inhibitory effect of RevErb on Bmal1 transcription which thus allows for light entrainment of Bmal1. Within the three node network of Per, Bmal1 and RevErb, all direct links mediated through cis regulatory elements are considered, see Fig 5D for a schematic drawing.

Values for all parameters have been obtained from [31] and modified manually in order to adapt simulated dynamics to experimental time series data as used throughout this study. The parameter values used in our numerical simulations are *d*_*P*_ = 0.25 h^−1^, *d*_*B*_ = 0.26 h^−1^, *d*_*R*_ = 0.29 h^−1^, *v*_*P*_ = 1, *v*_*B*_ = 0.9, *v*_*R*_ = 0.6, *k*_*P*_ = 0.1, *k*_*B*_ = 0.05, *k*_*R*_ = 0.9, *c*_*P*_ = 0.1, *c*_*R*_ = 35, *b*_*P*_ = 1, *b*_*R*_ = 8, $T_{P}=8.333\;\text{h}$, $T_{R}=1.52\;\text{h}$, and $T_{B}=3.652\;\text{h}$ unless otherwise stated. Hill coefficients are based upon experimentally observed binding sites as described in [31].

### Numerics

Simulations results in Figs 2E, 3, 4B and S5 Fig have been obtained by numerically solving the ordinary differential Eqs (1)–(3) via the odeint function from the integrate module of the Scientific Python package. The solutions have been drawn at equidistant intervals of Δ*t* = 0.01 h.

Simulations underlying S6 Fig are performed as described in the previous paragraph, after transforming Eq (9) into Cartesian coordinates.

Simulation results from the delay differential Eqs (10)–(13) as seen in Figs 5, 6, S7 and S9 Figs have been obtained numerically by means of the Matlab function dde23, called from a Python script using the matlab.engine API. Again, the solutions have been drawn at equidistant intervals of Δ*t* = 0.01h.

## Supporting information

S1 Fig Surrogate data generation. Depicted are various steps to generate the surrogate data as described in Section *Materials and Methods* of the *Main Text*. A) *N* cells are randomly located into a square shaped space from a two-dimensional uniform distribution. B) To each cellular position, an oscillating, sinusoidal intensity signal of period *τ*_*i*_ and initial phase *ϕ*_*i*_ is assigned. To mimick the experiment, periods and initial phases of *in silico* Bmal1 or Per1 signals are set differently. At each time point *t*, the signal is convoluted with a *Gaussian* kernel of standard deviation *σ*_*G*_ in order to mimic the spatial extension of neurons. C) *Gaussian* noise of standard deviation *σ*_*n*_ is independently added to the value of each pixel, at each time point *t*. D) Illustrative sketch of the resulting surrogate data image stack for exemplary time points. E) Example of individual surrogate time series data from a single pixel for both Bmal1 (orange) and Per1 (blue) image stacks.
(TIF)

S2 FigImpact of different *Gaussian* kernel width (*σ*_*G*_) on qualitative dynamical features, based on two hypotheses for surrogate data generation.*Top:* Example images of the Per1 surrogate time lapse movies at time point *t* = 0. Broadness of the *Gaussian* convolution kernels are increased from *left* to *right*, which can be associated with increasing neuron sizes or signal diffraction. Parameters *σ*_0_ = 0.0176, *N* = 150 and *σ*_*n*_ = 1 have been used. A standard deviation *σ*_*G*_ = *σ*_0_ of the *Gaussian* convolution kernel in the surrogate data generation approximates the size of an SCN neuron as recorded by the methods used in [21]. *Middle:* *Gaussian* kernel density estimates in the bivariate graph of Bmal1 and Per1 oscillation periods, estimated by a Lomb Scargle analysis of surrogate time lapse movies, generated under hypothesis $H_{0}^{\left(1\right)}$, *i.e.*, dynamical dissociation at the single cell level, for an increasing *Gaussian* kernel width (*σ*_*G*_) from left to right column. *Bottom:* Same as in the *middle* panel in case of hypothesis $H_{0}^{\left(2\right)}$, *i.e.*, randomly located cells with either a Bmal1 or Per1 signal of different periods.(TIF)

S3 FigEstimation of oscillatory parameters by cosine fitting.*Bmal1*, *RevErbα* and *Per1* gene expression profiles of the SCN tissue data set from [33] have been first normalized by their mean expression value (such that all profiles oscillate around the value of one) and then fitted by a simple harmonic function $y_{i}(t)=1+\left(a_{i}\text{cos}(\frac{2\pi }{\tau_{i}}t)+b_{i}\text{sin}(\frac{2\pi }{\tau_{i}}t)\right)$. Here, indices {*i*} denote fits to different time series of the three investigated clock genes. In panel A we allow individual periods *τ*_*i*_ for all three clock genes, while in panel B we assume a synchronized state between all clock genes such that the oscillation period *τ*_*i*_ ≕ *τ* is shared throughout the fit to all three clock genes. The fitted relative amplitudes and phases of the individual clock gene expression rhythms are given by $A_{rel,i}=\sqrt{a_{i}^{2}+b_{i}^{2}}$ and *ϕ*_*i*_ = arctan 2(*b*_*i*_, *a*_*i*_), respectively.(TIF)

S4 Fig Phase differences between Per and Bmal-Rev loops in case of synchronization. Borders of synchronization (bold black lines, see Inequality (4)) and color coded phase differences (see colorbar and Eq (8)) are plotted for the conceptual phase oscillator model as given by Eqs (1) and (2) of the *Main text* for different values of *β*. Δ*θ*^⋆^ ≈ −0.7 *π* denotes the experimentally observed phase differences between *Per1* and *Bmal1* gene oscillations as estimated from the SCN tissue data of [33], see also S3 Fig. Isoclines of a constant phase differences that match the experimentally observed value of Δ*θ*^⋆^ ≈ = −0.7*π* in the *K*-(*τ*^⋆^ − *τ*_*R*_) parameter plane are depicted by dashed white lines. These isoclines correpsond to the color-coded isoclines of Fig 2C of the *Main text*.
(TIF)

S5 Fig *Zeitgeber* intensity and inter-loop coupling determine jet-lag behavior. A) The Per loop dynamics shows a faster response to a 6h jet lag as the *Zeitgeber* intensity *z* is increased. Dynamics of the Bmal-Rev loop follow these dynamics although at a lower degree. B) Coupling constant *K* mainly determines how fast dynamics of the Bmal-Rev loop follow the relatively fast response of the Per loop to a 6h jet-lag. Response of the Per loop to jet-lag gets slower to some extent, since its dynamics is attracted to the Bmal-Rev loop by the symmetric coupling, which weakens the impact of *Zeitgeber* signal. C) Asymmetry in the coupling between the Per and Bmal-Rev loop has been introduced for a constant overall coupling strength *K* = *K*_*R*_ + *K*_*P*_ = *pK* + (1 − *p*)*K* by means of the asymmetry constant 0 ≤ *p* ≤ 1. Note that for *p* = 0 the system of coupled oscillators forms a chain, i.e., *Zeitgeber* signal *Z*(*t*) entrains the Per loop which in turn entrains the Bmal-Rev loop without any feedback from the Bmal-Rev to the Per loop. The coupling constant has been set to its nominal value of *K* ≈ 0.043 as determined in Fig 3A of the Main text. As long as synchronization between the Per and Bmal-Rev loop is achieved, a weaker impact of the Per onto the Bmal-Rev loop for *p* > 0.5 leads to a longer time of transient dynamical dissociation, eventually taking more than two weeks for the re-synchronization process, e.g., for *p* = 0.7.
(TIF)

S6 FigSelf-sustained as well as damped oscillations within a conceptual amplitude-phase model of the Bmal-Rev loop can account for the experimentally observed dynamics in case of weak inter-loop coupling.A) Schematic drawing of the conceptual model, comprised of two coupled Poincaré oscillators, representing autonomously oscillating Per and Bmal-Rev loops, where only the Per loop is directly driven by light. B) Region of synchronization between the Per and Bmal-Rev oscillators in the coupling strength *K* and period detuning parameter plane. Period detuning has been defined as the difference between the experimentally observed oscillation period *τ*^⋆^ ≈ 24.53h and the period *τ*_*P*_ of the Per loop. For the sake of simplicity a symmetric detuning of the Bmal-Rev loop from *τ*_⋆_ such that $\frac{\tau_{P}+\tau_{R}}{2}=\tau_{⋆}$ has been assumed, i.e. a period detuning of -1h translates to individual oscillator periods of *τ*_*P*_ = *τ*_⋆_ − 1*h* and *τ*_*R*_ = *τ*_⋆_ + 1*h*, respectively. As in S4 Fig, the dashed white line denotes parameter combinations whose synchronized dynamics exhibit the experimentally observed phase differences *θ*_⋆_ between the Per and Bmal1 oscillations. C) Similar to Fig 3A of the Main Text, the residual sum of squares (RSS) between simulated and experimentally observed jet-lag dynamics have been determined in the coupling strength *K* and *Zeitgeber* strength *z* parameter plane. For each *K*, a period detuning value from the dashed white line in panel B has been assigned such that the experimentally observed phase difference is conserved. D) Simulated (bold lines) free running (*z* = 0) oscillations of Per (blue) and Bmal1 (orange) for the optimal parameter set as depicted by the dashed black circle in panel B in comparison to corresponding experimental time series (dashed lines). E) A good agreement between simulated (bold lines) and experimentally obtained (dots) dynamics after a 6h phase advancing jet-lag can be observed for the optimal parameter set in panel B. Parameters underlying simulations in panel (B)-(E) are *A*_*P*_ = *A*_*R*_ = 1, λ_*P*_ = λ_*R*_ = 0.1*h*^−1^ and *ϕ* = *π*, compare Eq (9) of Section *Materials and Methods*. F-J) Same as in panels (A)-(E) in case of a damped Bmal-Rev loop, i.e. *A*_*R*_ has been set to zero in Eq (9) of Section *Materials and Methods*.(TIF)

S7 Fig Simulated dynamics of the single- and three-gene model under entrainment conditions. A) Single-gene model. B) Three gene model. For a *Zeitgeber* intensity of *z* = 0.21 that faithfully reproduces the experimentally observed response to a 6h phase advancing jet-lag, phases of entrainment of simulated Per, Bmal1, and RevErb gene expressions qualitatively coincide with those observed in experiments. While Per and RevErb show peaks around midday, Bmal1 shows morning peaks under LD12:12 equinoctial entrainment conditions.
(TIF)

S8 Fig Simulated dynamics of the three-gene model in state space. A) Simulated dynamics of the three gene model after a 9h light pulse in the three-dimensional state space (blue curve), corresponding to simulations depicted in Fig 6 of the main text. The black curve corresponds to the steady state limit cycle after transients decayed. B-D) Two-dimensional projections of the simulated dynamics shown in panel A.
(TIF)

S9 Fig Transient dissociation for weak coupling between Per and Bmal-Rev loops in the three-gene model. A) Analogously to Fig 6C of the *Main text*, simulated acrophases of Per (blue) and Bmal1 (orange, red, green) gene expressions, subject to a 9h light pulse, are depicted for different parameter values of *c*_*R*_. In case of Bmal1 oscillations, simulations with different values of *c*_*R*_ are highlighted by different marker symbols and colors. Long lasting transient dissociation dynamics (more than two weeks) can be observed for large values of *c*_*R*_ which corresponds to a weak coupling between the Per and Bmal-Rev loop due to a reduced transcriptional repression of Rev by Per. B) Instantaneous periods as determined from the time differences between two consecutive acrophases in panel A. In dependence on the constant *c*_*R*_, either longer or shorter instantaneous Bmal1 periods compared to Per oscillation periods can be observed after a 9h light pulse. C) Analogously to panel (A), simulated acrophases of Per (blue) and Bmal1 (orange, green, black) gene expressions after a 6h phase advancing jet-lag are depicted for different parameter values of *c*_*R*_. D) Again, varying parameters of *c*_*R*_ lead to different re-entrainment times, ultimately leading to differing values of instantaneous periods of the Per (blue) and Bmal1 (orange, green, black) gene expressions.
(TIF)

S10 Fig “Coupling” between feedback loops determines time scale of transient dissociation in the three-gene model. A) Phase response curves (PRCs) of the three gene model, determined for 9h *Zeitgeber* pulses (*z* = 0.43) applied to the Per variable at different times around subjective day. PRCs have been determined for different parameters values *c*_*R*_ that can be associated with the impact (coupling strength) of the Per onto the Bmal-Rev loop. It can be noted that the PRC is barely affected by the different values of *c*_*R*_. B-D) Time to re-entrain for the *Zeitgeber* pulses as described for panel (A) in case of Per (B), Bmal1 (C), or Rev-Erb (D) for different values of *c*_*R*_. While the generally shorter re-entrainment time of Per barely changes with alterations in *c*_*R*_, the re-entrainment time of Bmall1 and Rev-Erb increases with increasing *c*_*R*_ (decreasing coupling between the Per and Bmal-Rev loops). E) PRCs of the three gene model as in panel (A), determined for 9h *Zeitgeber* pulses (*z* = 4.3) applied to the Rev-Erb variable (in the same way as described for the Per variable in Eq (11) of the Main text) for different parameters values *b*_*P*_ that can be associated with the impact (coupling strength) of the Bmal-Rev onto the Per loop. F-H) Re-entrainment time, analogously to panels (B)-(D) in case of different values for *b*_*P*_ and a *Zeitgeber* signal applied to Rev-Erb. Conclusively, one can observe that a wide range of re-entrainment times are possible in dependence of the phase of the *Zeitgeber* pulse as well as the parameter values associated with inter-loop “coupling”.
(TIF)

S11 Fig Transient dissociation can be observed within a larger mammalian core clock model. A) Schematic drawing of the regulatory core clock network. In this model, the network of 20 known clock genes has been condensed into gene regulatory interactions of five groups of genes, see table in panel (A) and references [39, 40]. B) Simulation of the *Per*, *Bmal1*, and *RevErb* genes (top, bold lines) as well as the corresponding experimental time series (bottom, dashed lines) from SCN tissue as obtained from the high throughput study in reference [33]. Please note that kinetic parameters have been fitted to account for experimental *Per2* time series data as done in [39, 40]. This results in a later phase of simulated *Per* free-running gene expressions in comparison to the conceptual phase oscillator and the three-gene model, where kinetic parameters have been optimized to account for experimental *Per1* gene expressions. C) Simulated dynamics under equinoctial LD12:12 entrainment conditions for a *Zeitgeber* intensity of *z* = 0.015. D) Simulated differential responses to a 6h phase advancing jet-lag between Per and Bmal-Rev loops together with the corresponding experimental data for *Per2*, *Bmal1*, and *RevErbα* genes.
(TIF)

S1 Text Detailed information on the molecular five variable model, the corresponding parameter fitting procedure as well as a representative set of parameters. (PDF)
