# Supplementary figures and images for: Weak coupling between intracellular feedback loops explains dissociation of clock gene dynamics
Source: PLoS Comput Biol. 2019 Sep 12;15(9):e1007330. doi: 10.1371/journal.pcbi.1007330 (PMC6759184; doi:10.1371/journal.pcbi.1007330)

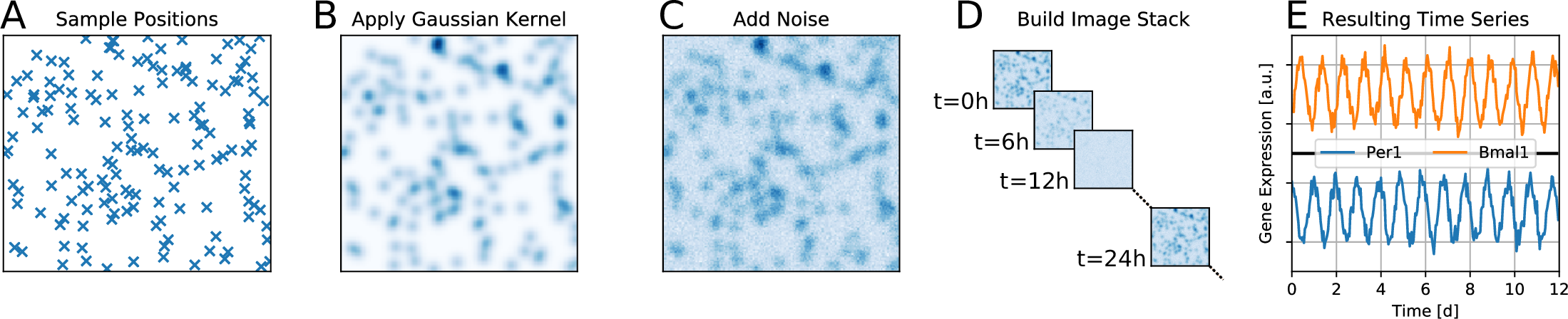

Supplement: S1 Fig — Depicted are various steps to generate the surrogate data as described in Section Materials and Methods of the Main Text. A) N cells are randomly located into a square shaped space from a two-dimensional uniform distribution. B) To each cellular position, an oscillating, sinusoidal intensity signal of period τi and initial phase ϕi is assigned. To mimick the experiment, periods and initial phases of in silico Bmal1 or Per1 signals are set differently. At each time point t, the signal is convoluted with a Gaussian kernel of standard deviation σG in order to mimic the spatial extension of neurons. C) Gaussian noise of standard deviation σn is independently added to the value of each pixel, at each time point t. D) Illustrative sketch of the resulting surrogate data image stack for exemplary time points. E) Example of individual surrogate time series data from a single pixel for both Bmal1 (orange) and Per1 (blue) image stacks. (TIF) [file pcbi.1007330.s001.tif]

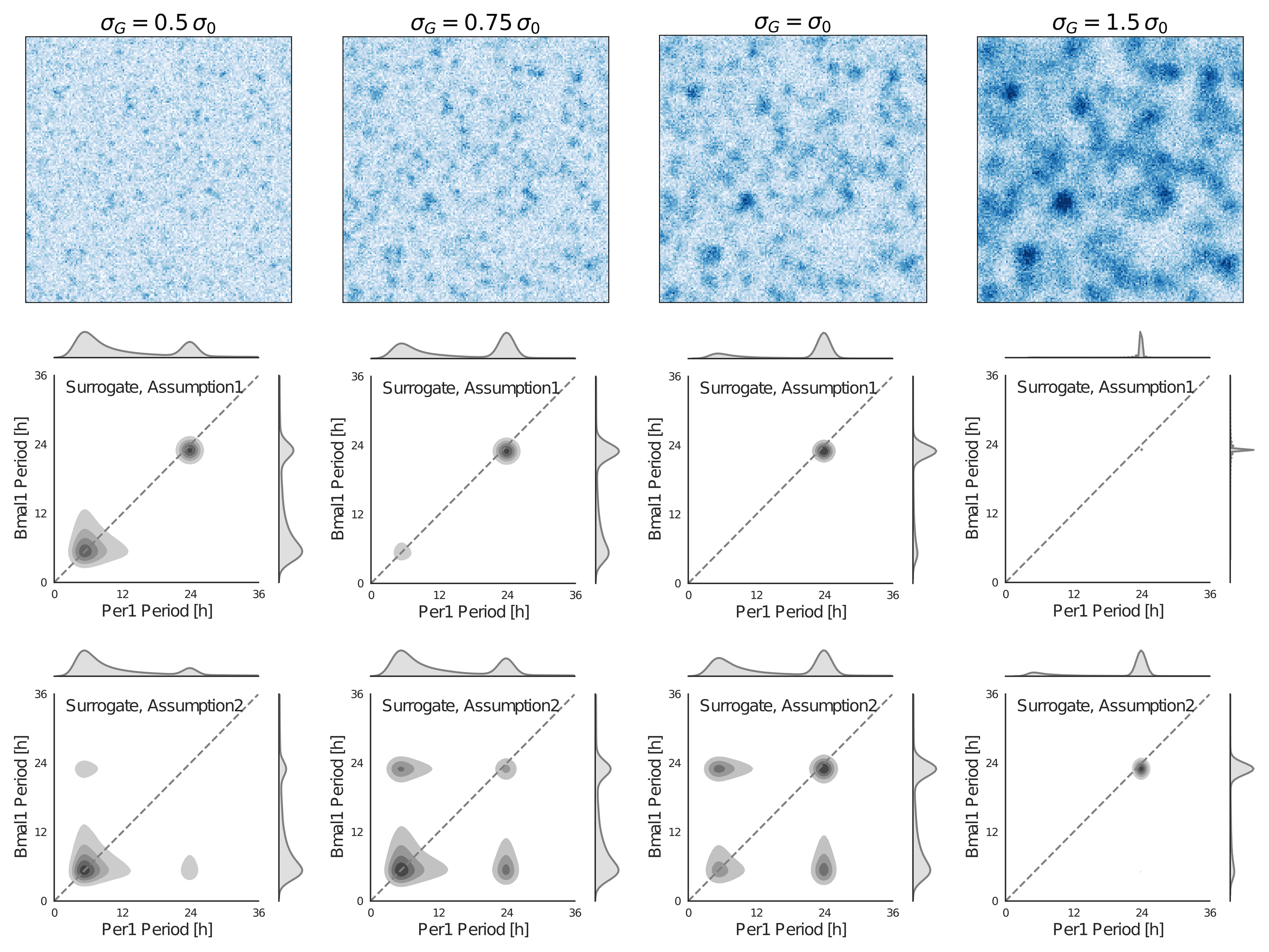

Supplement: S2 Fig — Top: Example images of the Per1 surrogate time lapse movies at time point t = 0. Broadness of the Gaussian convolution kernels are increased from left to right, which can be associated with increasing neuron sizes or signal diffraction. Parameters σ0 = 0.0176, N = 150 and σn = 1 have been used. A standard deviation σG = σ0 of the Gaussian convolution kernel in the surrogate data generation approximates the size of an SCN neuron as recorded by the methods used in [21]. Middle: Gaussian kernel density estimates in the bivariate graph of Bmal1 and Per1 oscillation periods, estimated by a Lomb Scargle analysis of surrogate time lapse movies, generated under hypothesis H0(1), i.e., dynamical dissociation at the single cell level, for an increasing Gaussian kernel width (σG) from left to right column. Bottom: Same as in the middle panel in case of hypothesis H0(2), i.e., randomly located cells with either a Bmal1 or Per1 signal of different periods. (TIF) [file pcbi.1007330.s002.tif]

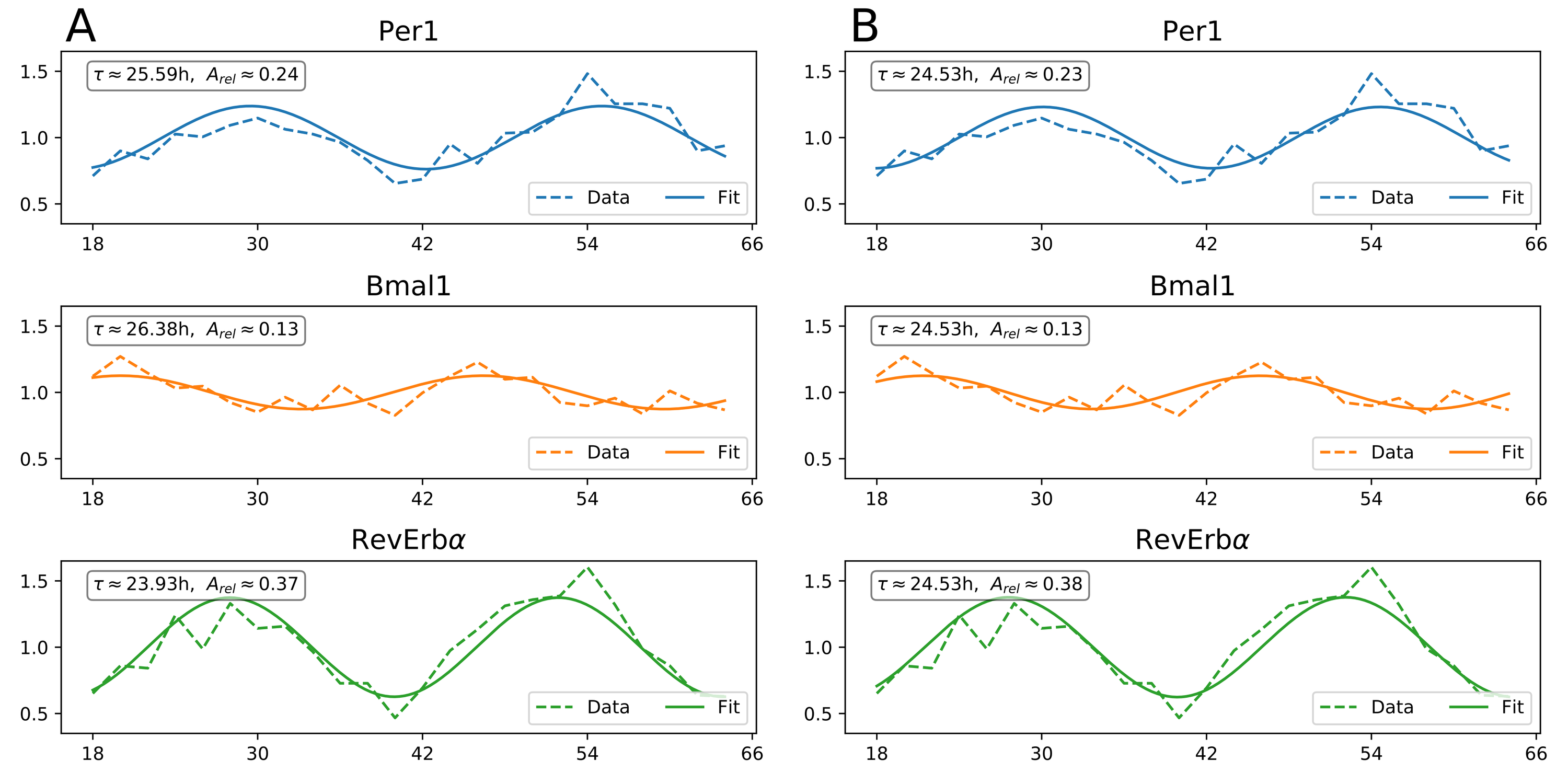

Supplement: S3 Fig — Bmal1, RevErbα and Per1 gene expression profiles of the SCN tissue data set from [33] have been first normalized by their mean expression value (such that all profiles oscillate around the value of one) and then fitted by a simple harmonic function yi(t)=1+(aicos(2πτit)+bisin(2πτit)). Here, indices {i} denote fits to different time series of the three investigated clock genes. In panel A we allow individual periods τi for all three clock genes, while in panel B we assume a synchronized state between all clock genes such that the oscillation period τi ≕ τ is shared throughout the fit to all three clock genes. The fitted relative amplitudes and phases of the individual clock gene expression rhythms are given by Arel,i=ai2+bi2 and ϕi = arctan 2(bi, ai), respectively. (TIF) [file pcbi.1007330.s003.tif]

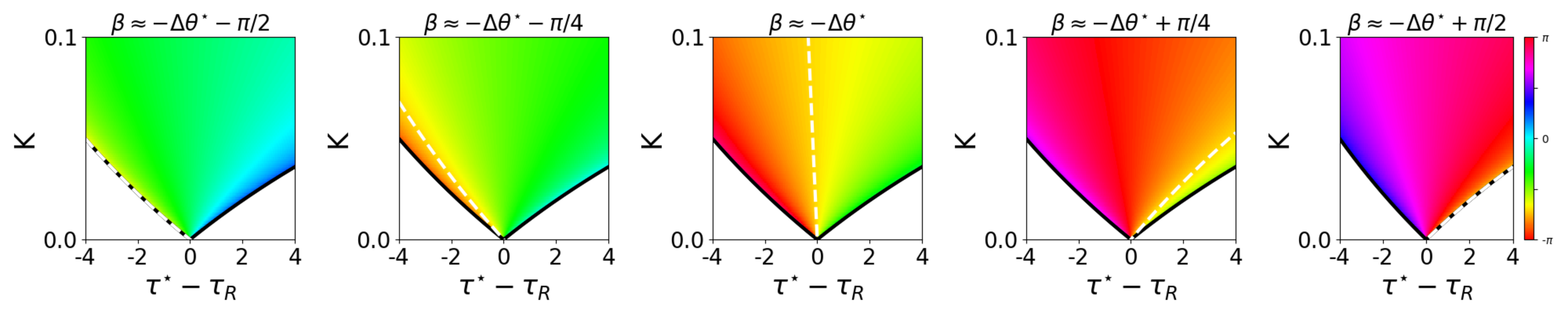

Supplement: S4 Fig — Borders of synchronization (bold black lines, see Inequality (4)) and color coded phase differences (see colorbar and Eq (8)) are plotted for the conceptual phase oscillator model as given by Eqs (1) and (2) of the Main text for different values of β. Δθ⋆ ≈ −0.7 π denotes the experimentally observed phase differences between Per1 and Bmal1 gene oscillations as estimated from the SCN tissue data of [33], see also S3 Fig. Isoclines of a constant phase differences that match the experimentally observed value of Δθ⋆ ≈ = −0.7π in the K-(τ⋆ − τR) parameter plane are depicted by dashed white lines. These isoclines correpsond to the color-coded isoclines of Fig 2C of the Main text. (TIF) [file pcbi.1007330.s004.tif]

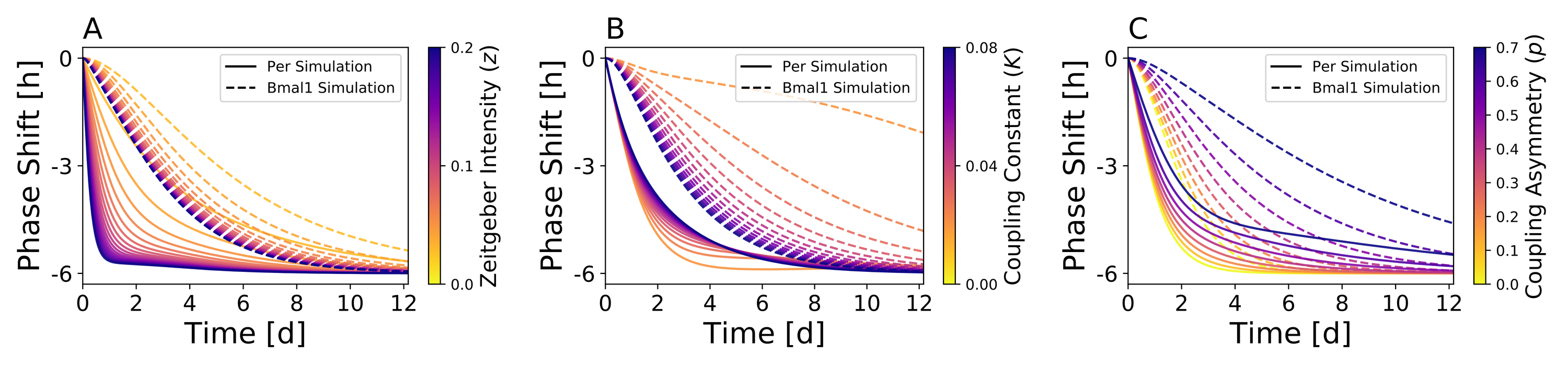

Supplement: S5 Fig — A) The Per loop dynamics shows a faster response to a 6h jet lag as the Zeitgeber intensity z is increased. Dynamics of the Bmal-Rev loop follow these dynamics although at a lower degree. B) Coupling constant K mainly determines how fast dynamics of the Bmal-Rev loop follow the relatively fast response of the Per loop to a 6h jet-lag. Response of the Per loop to jet-lag gets slower to some extent, since its dynamics is attracted to the Bmal-Rev loop by the symmetric coupling, which weakens the impact of Zeitgeber signal. C) Asymmetry in the coupling between the Per and Bmal-Rev loop has been introduced for a constant overall coupling strength K = KR + KP = pK + (1 − p)K by means of the asymmetry constant 0 ≤ p ≤ 1. Note that for p = 0 the system of coupled oscillators forms a chain, i.e., Zeitgeber signal Z(t) entrains the Per loop which in turn entrains the Bmal-Rev loop without any feedback from the Bmal-Rev to the Per loop. The coupling constant has been set to its nominal value of K ≈ 0.043 as determined in Fig 3A of the Main text. As long as synchronization between the Per and Bmal-Rev loop is achieved, a weaker impact of the Per onto the Bmal-Rev loop for p > 0.5 leads to a longer time of transient dynamical dissociation, eventually taking more than two weeks for the re-synchronization process, e.g., for p = 0.7. (TIF) [file pcbi.1007330.s005.tif]

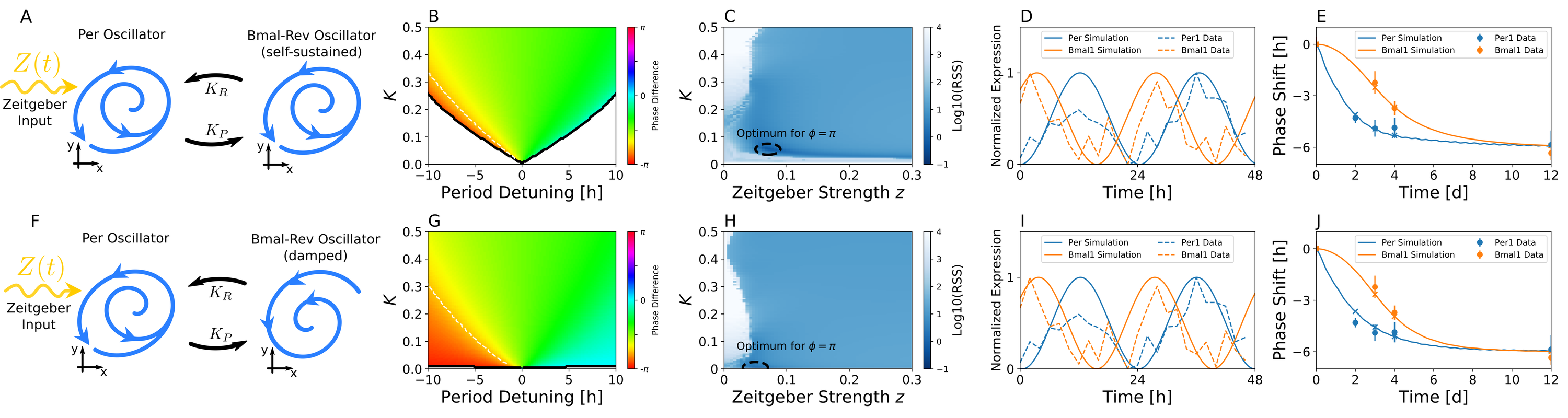

Supplement: S6 Fig — A) Schematic drawing of the conceptual model, comprised of two coupled Poincaré oscillators, representing autonomously oscillating Per and Bmal-Rev loops, where only the Per loop is directly driven by light. B) Region of synchronization between the Per and Bmal-Rev oscillators in the coupling strength K and period detuning parameter plane. Period detuning has been defined as the difference between the experimentally observed oscillation period τ⋆ ≈ 24.53h and the period τP of the Per loop. For the sake of simplicity a symmetric detuning of the Bmal-Rev loop from τ⋆ such that τP+τR2=τ⋆ has been assumed, i.e. a period detuning of -1h translates to individual oscillator periods of τP = τ⋆ − 1h and τR = τ⋆ + 1h, respectively. As in S4 Fig, the dashed white line denotes parameter combinations whose synchronized dynamics exhibit the experimentally observed phase differences θ⋆ between the Per and Bmal1 oscillations. C) Similar to Fig 3A of the Main Text, the residual sum of squares (RSS) between simulated and experimentally observed jet-lag dynamics have been determined in the coupling strength K and Zeitgeber strength z parameter plane. For each K, a period detuning value from the dashed white line in panel B has been assigned such that the experimentally observed phase difference is conserved. D) Simulated (bold lines) free running (z = 0) oscillations of Per (blue) and Bmal1 (orange) for the optimal parameter set as depicted by the dashed black circle in panel B in comparison to corresponding experimental time series (dashed lines). E) A good agreement between simulated (bold lines) and experimentally obtained (dots) dynamics after a 6h phase advancing jet-lag can be observed for the optimal parameter set in panel B. Parameters underlying simulations in panel (B)-(E) are AP = AR = 1, λP = λR = 0.1h−1 and ϕ = π, compare Eq (9) of Section Materials and Methods. F-J) Same as in panels (A)-(E) in case of a damped Bmal-Rev loop, i.e. AR has been set to zero in Eq (9) of Sec [file pcbi.1007330.s006.tif]

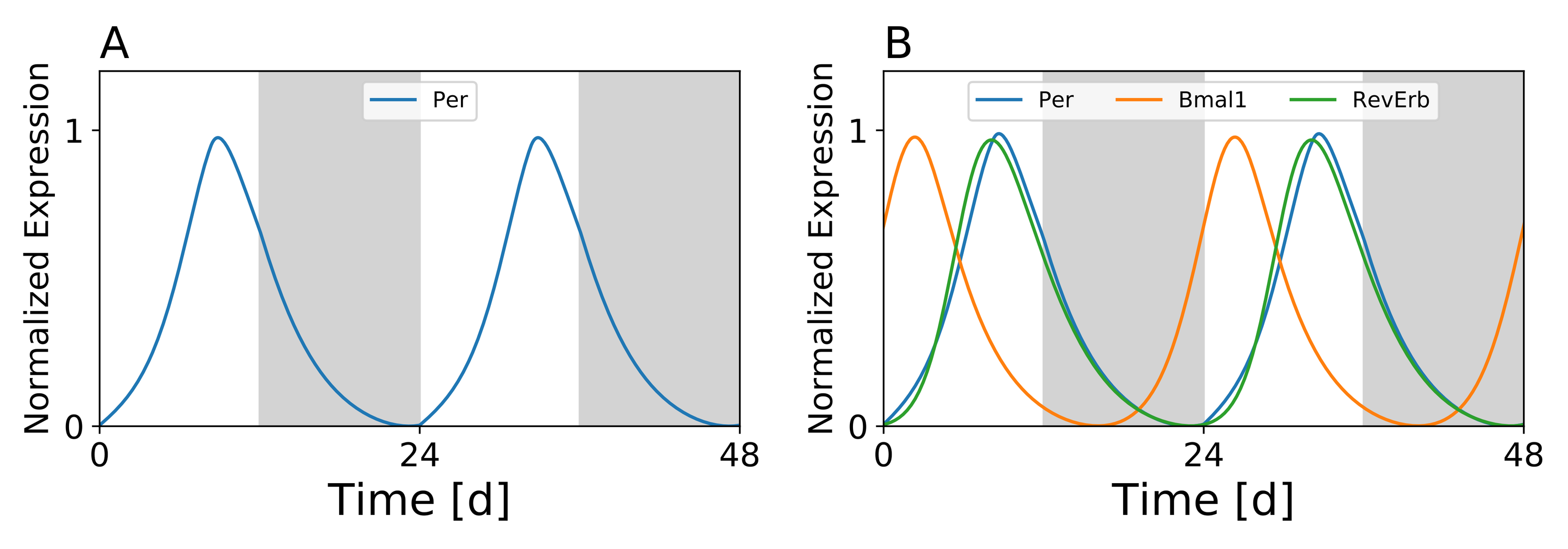

Supplement: S7 Fig — A) Single-gene model. B) Three gene model. For a Zeitgeber intensity of z = 0.21 that faithfully reproduces the experimentally observed response to a 6h phase advancing jet-lag, phases of entrainment of simulated Per, Bmal1, and RevErb gene expressions qualitatively coincide with those observed in experiments. While Per and RevErb show peaks around midday, Bmal1 shows morning peaks under LD12:12 equinoctial entrainment conditions. (TIF) [file pcbi.1007330.s007.tif]

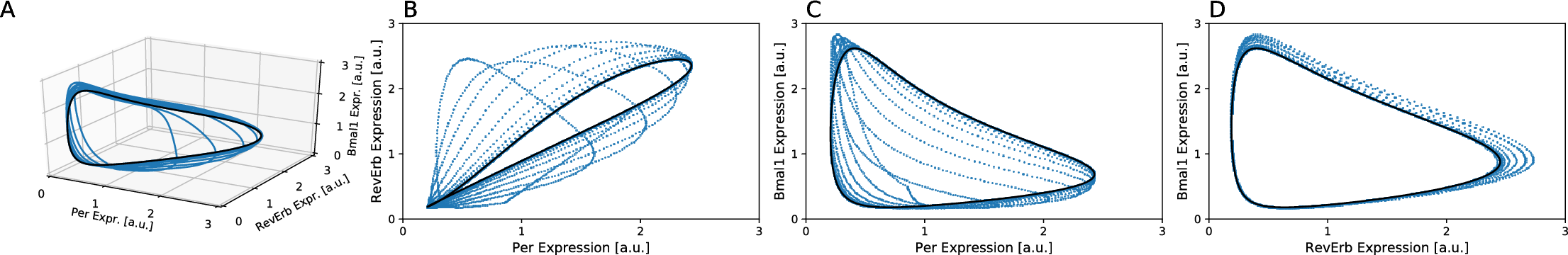

Supplement: S8 Fig — A) Simulated dynamics of the three gene model after a 9h light pulse in the three-dimensional state space (blue curve), corresponding to simulations depicted in Fig 6 of the main text. The black curve corresponds to the steady state limit cycle after transients decayed. B-D) Two-dimensional projections of the simulated dynamics shown in panel A. (TIF) [file pcbi.1007330.s008.tif]

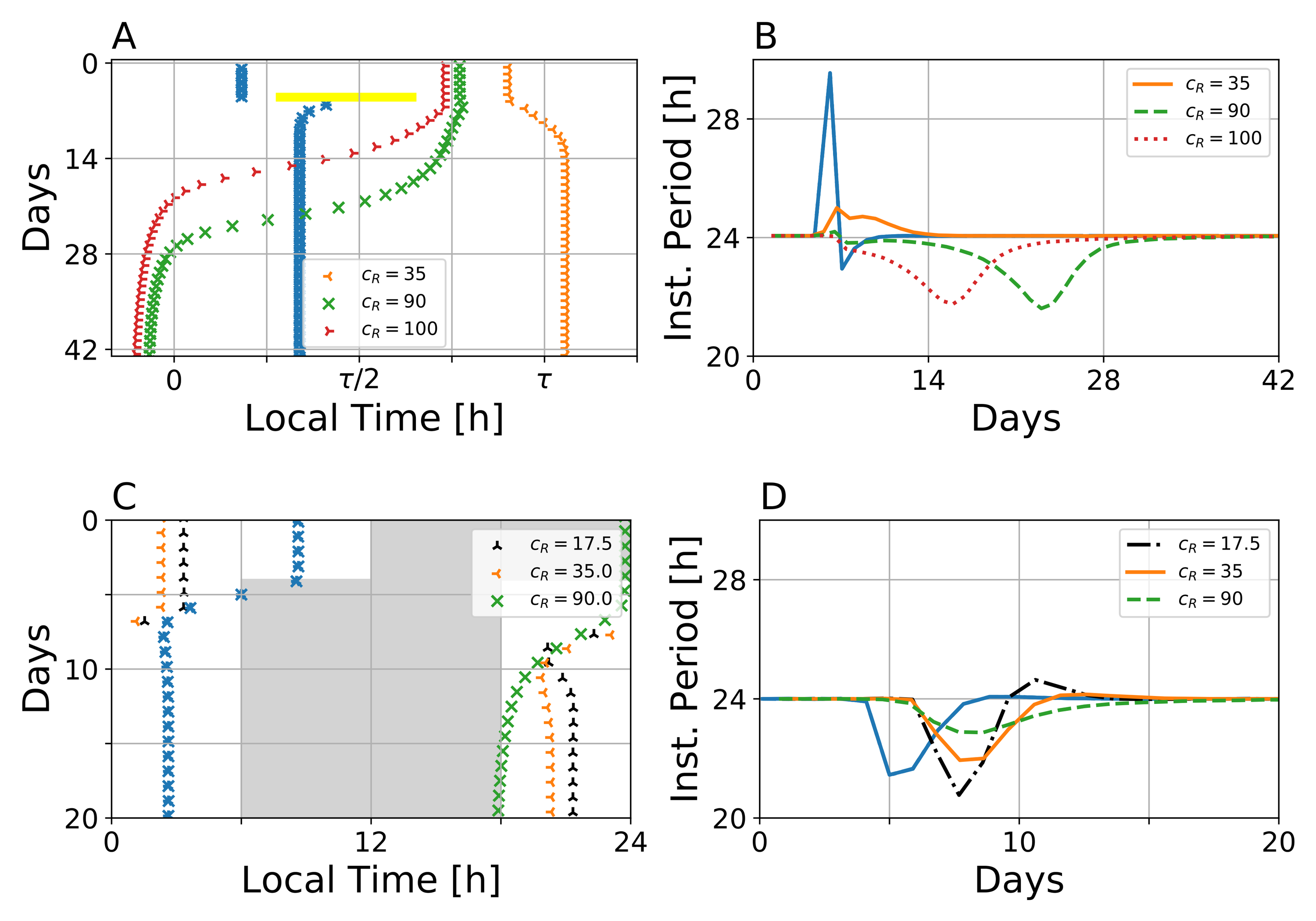

Supplement: S9 Fig — A) Analogously to Fig 6C of the Main text, simulated acrophases of Per (blue) and Bmal1 (orange, red, green) gene expressions, subject to a 9h light pulse, are depicted for different parameter values of cR. In case of Bmal1 oscillations, simulations with different values of cR are highlighted by different marker symbols and colors. Long lasting transient dissociation dynamics (more than two weeks) can be observed for large values of cR which corresponds to a weak coupling between the Per and Bmal-Rev loop due to a reduced transcriptional repression of Rev by Per. B) Instantaneous periods as determined from the time differences between two consecutive acrophases in panel A. In dependence on the constant cR, either longer or shorter instantaneous Bmal1 periods compared to Per oscillation periods can be observed after a 9h light pulse. C) Analogously to panel (A), simulated acrophases of Per (blue) and Bmal1 (orange, green, black) gene expressions after a 6h phase advancing jet-lag are depicted for different parameter values of cR. D) Again, varying parameters of cR lead to different re-entrainment times, ultimately leading to differing values of instantaneous periods of the Per (blue) and Bmal1 (orange, green, black) gene expressions. (TIF) [file pcbi.1007330.s009.tif]

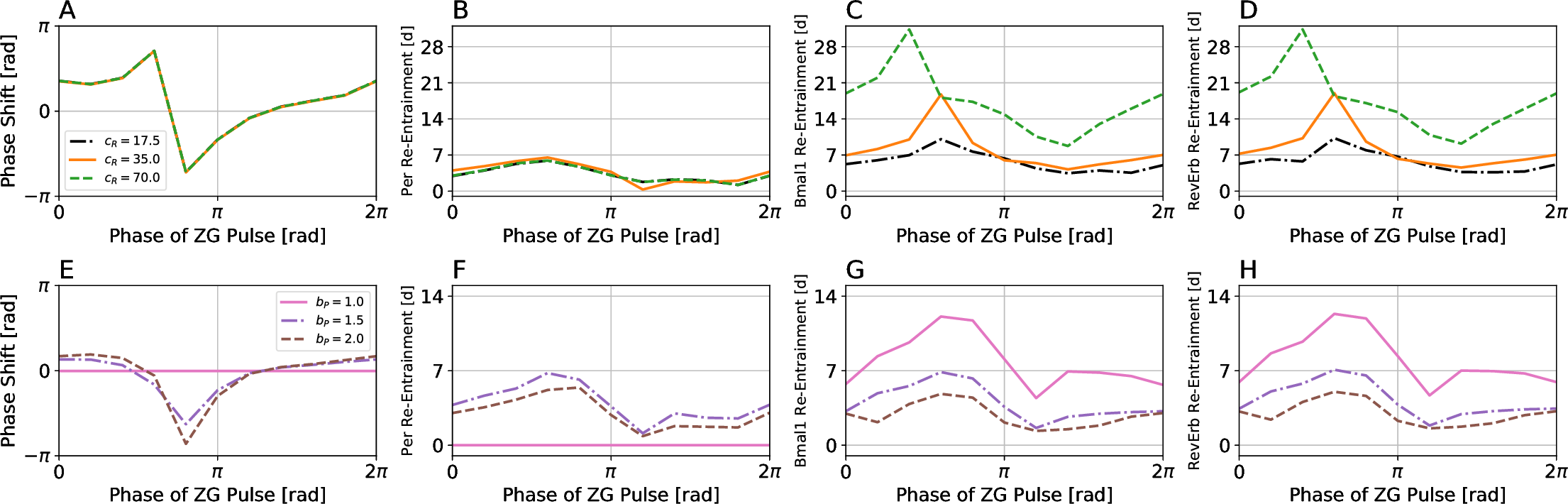

Supplement: S10 Fig — A) Phase response curves (PRCs) of the three gene model, determined for 9h Zeitgeber pulses (z = 0.43) applied to the Per variable at different times around subjective day. PRCs have been determined for different parameters values cR that can be associated with the impact (coupling strength) of the Per onto the Bmal-Rev loop. It can be noted that the PRC is barely affected by the different values of cR. B-D) Time to re-entrain for the Zeitgeber pulses as described for panel (A) in case of Per (B), Bmal1 (C), or Rev-Erb (D) for different values of cR. While the generally shorter re-entrainment time of Per barely changes with alterations in cR, the re-entrainment time of Bmall1 and Rev-Erb increases with increasing cR (decreasing coupling between the Per and Bmal-Rev loops). E) PRCs of the three gene model as in panel (A), determined for 9h Zeitgeber pulses (z = 4.3) applied to the Rev-Erb variable (in the same way as described for the Per variable in Eq (11) of the Main text) for different parameters values bP that can be associated with the impact (coupling strength) of the Bmal-Rev onto the Per loop. F-H) Re-entrainment time, analogously to panels (B)-(D) in case of different values for bP and a Zeitgeber signal applied to Rev-Erb. Conclusively, one can observe that a wide range of re-entrainment times are possible in dependence of the phase of the Zeitgeber pulse as well as the parameter values associated with inter-loop “coupling”. (TIF) [file pcbi.1007330.s010.tif]

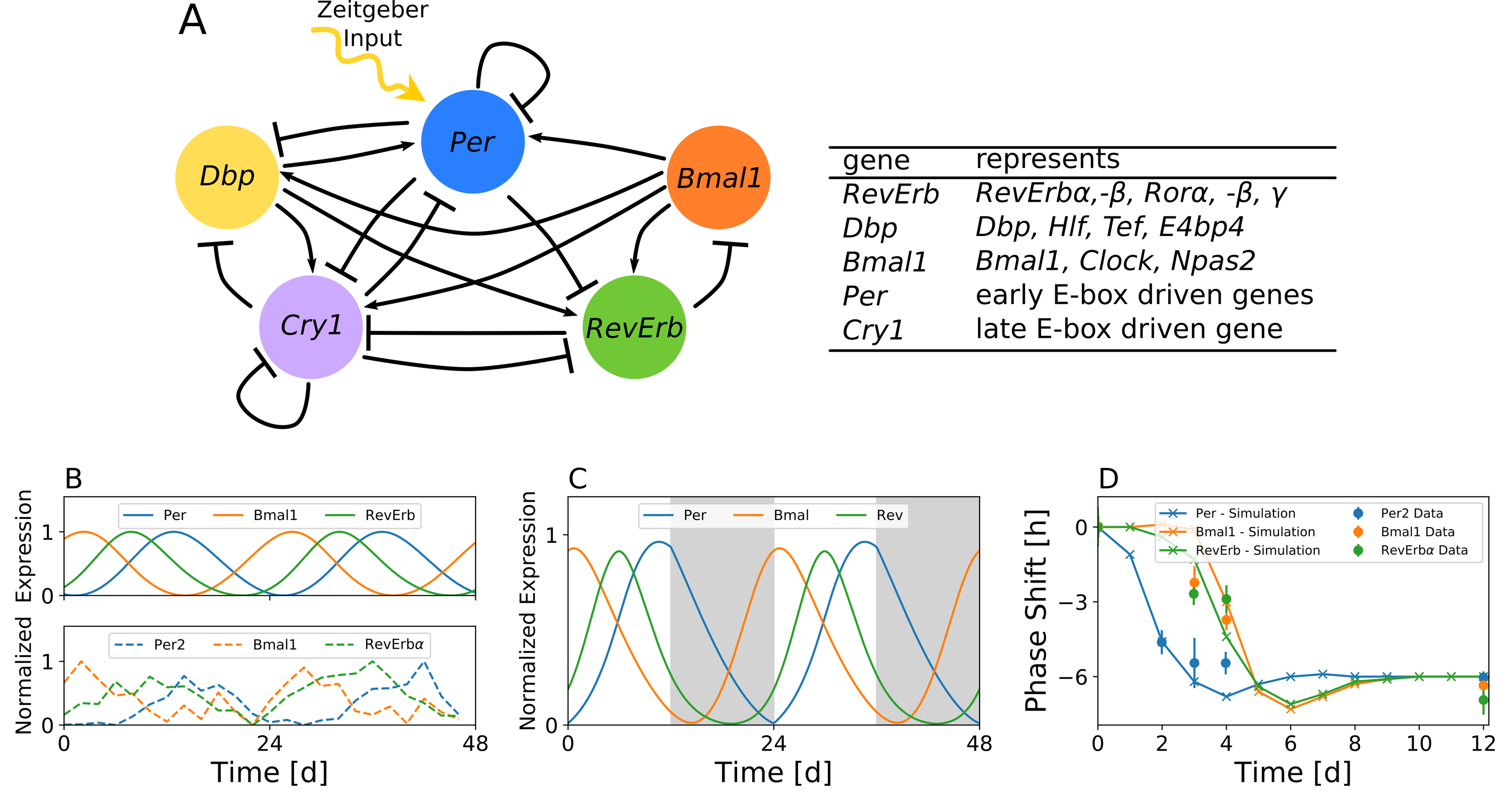

Supplement: S11 Fig — A) Schematic drawing of the regulatory core clock network. In this model, the network of 20 known clock genes has been condensed into gene regulatory interactions of five groups of genes, see table in panel (A) and references [39, 40]. B) Simulation of the Per, Bmal1, and RevErb genes (top, bold lines) as well as the corresponding experimental time series (bottom, dashed lines) from SCN tissue as obtained from the high throughput study in reference [33]. Please note that kinetic parameters have been fitted to account for experimental Per2 time series data as done in [39, 40]. This results in a later phase of simulated Per free-running gene expressions in comparison to the conceptual phase oscillator and the three-gene model, where kinetic parameters have been optimized to account for experimental Per1 gene expressions. C) Simulated dynamics under equinoctial LD12:12 entrainment conditions for a Zeitgeber intensity of z = 0.015. D) Simulated differential responses to a 6h phase advancing jet-lag between Per and Bmal-Rev loops together with the corresponding experimental data for Per2, Bmal1, and RevErbα genes. (TIF) [file pcbi.1007330.s011.tif]
